# A comprehensive update on CIDO: the community-based coronavirus infectious disease ontology

**DOI:** 10.1186/s13326-022-00279-z

**Published:** 2022-10-21

**Authors:** Yongqun He, Hong Yu, Anthony Huffman, Asiyah Yu Lin, Darren A. Natale, John Beverley, Ling Zheng, Yehoshua Perl, Zhigang Wang, Yingtong Liu, Edison Ong, Yang Wang, Philip Huang, Long Tran, Jinyang Du, Zalan Shah, Easheta Shah, Roshan Desai, Hsin-hui Huang, Yujia Tian, Eric Merrell, William D. Duncan, Sivaram Arabandi, Lynn M. Schriml, Jie Zheng, Anna Maria Masci, Liwei Wang, Hongfang Liu, Fatima Zohra Smaili, Robert Hoehndorf, Zoë May Pendlington, Paola Roncaglia, Xianwei Ye, Jiangan Xie, Yi-Wei Tang, Xiaolin Yang, Suyuan Peng, Luxia Zhang, Luonan Chen, Junguk Hur, Gilbert S. Omenn, Brian Athey, Barry Smith

**Affiliations:** 1 University of Michigan Medical School, Ann Arbor, MI USA; 2 People’s Hospital of Guizhou Province, Guiyang, Guizhou China; 3 National Human Genome Research Institute, National Institutes of Health, Bethesda, MD USA; 4 National Center for Ontological Research, Buffalo, NY USA; 5 Georgetown University Medical Center, Washington, DC USA; 6 The Johns Hopkins University Applied Physics Laboratory, Laurel, MD USA; 7 Computer Science and Software Engineering Department, Monmouth University, West Long Branch, NJ USA; 8 Department of Computer Science, New Jersey Institute of Technology, Newark, NJ USA; 9 Institute of Basic Medical Sciences, Chinese Academy of Medical Sciences & School of Basic Medicine, Peking Union Medical College, Beijing, China; 10 National Yang-Ming University, Taipei, Taiwan; 11 Rutgers University, New Brunswick, NJ USA; 12 University at Buffalo, Buffalo, NY 14260 USA; 13 University of Florida, Gainesville, FL USA; 14 OntoPro LLC, Houston, TX USA; 15 University of Maryland School of Medicine, Baltimore, MD USA; 16 Department of Biology, University of Pennsylvania Perelman School of Medicine, Philadelphia, PA USA; 17 Office of Data Science, National Institute of Environmental Health Sciences, Research Triangle Park, NC USA; 18 Mayo Clinic, Rochester, MN USA; 19 King Abdullah University of Science and Technology, Thuwal, Saudi Arabia; 20 European Bioinformatics Institute (EMBL-EBI), Wellcome Genome Campus, Hinxton, Cambridgeshire, UK; 21 School of Bioinformatics, Chongqing University of Posts and Telecommunications, Chongqing, China; 22 Cepheid, Danaher Diagnostic Platform, Shanghai, China; 23 National Institute of Health Data Science, Peking University, Beijing, China; 24 Shanghai Institute of Biochemistry and Cell Biology, Chinese Academy of Sciences, Shanghai, China; 25 University of North Dakota School of Medicine and Health Sciences, Grand Forks, ND USA

**Keywords:** Coronavirus, COVID-19, SARS-CoV-2, Ontology, Phenotype, Diagnosis, Vaccine, Drug repurposing

## Abstract

**Background:**

The current COVID-19 pandemic and the previous SARS/MERS outbreaks of 2003 and 2012 have resulted in a series of major global public health crises. We argue that in the interest of developing effective and safe vaccines and drugs and to better understand coronaviruses and associated disease mechenisms it is necessary to integrate the large and exponentially growing body of heterogeneous coronavirus data. Ontologies play an important role in standard-based knowledge and data representation, integration, sharing, and analysis. Accordingly, we initiated the development of the community-based Coronavirus Infectious Disease Ontology (CIDO) in early 2020.

**Results:**

As an Open Biomedical Ontology (OBO) library ontology, CIDO is open source and interoperable with other existing OBO ontologies. CIDO is aligned with the Basic Formal Ontology and Viral Infectious Disease Ontology. CIDO has imported terms from over 30 OBO ontologies. For example, CIDO imports all SARS-CoV-2 protein terms from the Protein Ontology, COVID-19-related phenotype terms from the Human Phenotype Ontology, and over 100 COVID-19 terms for vaccines (both authorized and in clinical trial) from the Vaccine Ontology. CIDO systematically represents variants of SARS-CoV-2 viruses and over 300 amino acid substitutions therein, along with over 300 diagnostic kits and methods. CIDO also describes hundreds of host-coronavirus protein-protein interactions (PPIs) and the drugs that target proteins in these PPIs. CIDO has been used to model COVID-19 related phenomena in areas such as epidemiology. The scope of CIDO was evaluated by visual analysis supported by a summarization network method. CIDO has been used in various applications such as term standardization, inference, natural language processing (NLP) and clinical data integration. We have applied the amino acid variant knowledge present in CIDO to analyze differences between SARS-CoV-2 Delta and Omicron variants. CIDO's integrative host-coronavirus PPIs and drug-target knowledge has also been used to support drug repurposing for COVID-19 treatment.

**Conclusion:**

CIDO represents entities and relations in the domain of coronavirus diseases with a special focus on COVID-19. It supports shared knowledge representation, data and metadata standardization and integration, and has been used in a range of applications.

**Supplementary Information:**

The online version contains supplementary material available at 10.1186/s13326-022-00279-z.

## Background

Coronavirus diseases pose major challenges to public health. In addition to the current Coronavirus Disease 2019 (COVID-19) pandemic, Severe Acute Respiratory Syndrome (SARS) [1] and Middle East Respiratory Syndrome (MERS) [2] are two other severe human coronavirus diseases that have arisen in the past two decades. The World Health Organization (WHO) declared the COVID-19 outbreak as a pandemic on March 11, 2020; at that time there were 118,326 confirmed cases and 4292 deaths globally [3]. As of April 27, 2022, the number of COVID-19 confirmed cases has risen to over 500 million confirmed cases, resulting in over 6 million deaths globally. The dramatic increase of COVID-19-related cases and deaths over 2 years illustrates the urgent need for collaborative research on coronavirus diseases, especially COVID-19, by researchers around the world.

Extensive COVID-19 research has been conducted since the start of the pandemic. For example, there have been over 250,000 COVID-19-related papers recorded in PubMed as of April 2022. These research articles cover various domains such as etiology, epidemiology, and biotechnology. The initial wave of research articles focused on characterization of the original Wuhan strain of SARS-CoV-2 [4], the molecular interactions of putative and confirmed SARS-CoV-2 molecules [5], and the unique disease phenotype of COVID-19 [6]. During this time, many novel and repurposed medical treatments were developed and authorized to treat or prevent COVID-19. This included research to develop effective COVID-19 vaccines [7] and COVID-19 drug treatments [8]. However, the emergence of new SARS-CoV-2 variants with unique traits prompted novel research investigating the fundamental molecular mechanisms of virulence and transmission associated with these variants [9].

Throughout the COVID-19 pandemic, epidemiological data from across the globe has been collected for viral sequences and human demographics. In the era of Information Technology and big data, biomedical research has become data-intensive with the generation of increasingly large, complex, multidimensional, and diverse datasets. The explosion of valuable data and knowledge related to COVID-19 fits the 5Vs of big data (volume, veracity, velocity, variety, and value) [10, 11] and represents a wealth of knowledge related to SARS-CoV-2. However, these studies are often stored in non-interoperable data repositories which resist integration, creating a major bottleneck for COVID-19 research. The resultant non-harmonized data and knowledge cannot be easily analyzed by standard Artificial Intelligence (AI)/Machine Learning (ML) techniques. The development of computer-interpretable, integrative, interoperable ontologies can contribute to needed data harmonization.

Such observations led to the development of a community-based, interoperable Coronavirus Infectious Disease Ontology (CIDO) for standardized and efficient representation, integration, and analysis of coronavirus disease data. CIDO was initiated by He and Yu in early 2020 when the COVID-19 became endemic in China. CIDO was accepted into the Open Biomedical Ontologies library in March 2020, and was initially reported in a *Comment* article in the journal *Scientific Data* [12]. In that article, CIDO was introduced as a community-driven open-source OBO library ontology providing standardized, computer-interpretable terminological content for various coronavirus infectious diseases, including their etiology, transmission, epidemiology, pathogenesis, host-coronavirus interactions, diagnosis, prevention, and treatment. Additionally, it was shown how host-coronavirus interaction mechanisms could be represented using CIDO resources and axioms, and how such representation could be used to aid in the identification of potential COVID-19 treatment options based on existing knowledge of drug mechanisms of action. Indeed, it was reported that CIDO provided instrumental guidance during literature mining processes in which 72 chemical drugs and 27 monoclonal or polyclonal antibodies that exhibit anti-coronavirus effects in in vitro or in vivo experimental studies were identified. The *Scientific Data* article closed by inviting researchers from across the world to contribute to CIDO development and application. We are pleased to report that there has been an outpouring of community support, and substantial CIDO development and application since that time.

CIDO was presented at the 2020 International Conference on Biomedical Ontology (ICBO-2020) [13]. Subsequently, authors AYL, YQH, SA, and WD organized a “Workshop on COVID-19 Ontologies” (WCO 2020) in October 2020 (https://github.com/CIDO-ontology/WCO), which led to the on-going harmonization of 9 COVID-19 related ontologies. Of these ontologies, CIDO subsumed the COVID-19 Infectious Disease Ontology (IDO-COVID-19) and initiated alignment with the Controlled Vocabulary for COVID-19 (COVoc). The ontology harmonization effort was also presented in ICBO-2021 [14]. Since then, CIDO has been further developed to include more terms and relations in many areas, such as host responses to SARS-CoV-2 infection [15], host-coronavirus protein-protein interactions, and COVID-19 diagnosis and vaccines. This journal manuscript provides a comprehensive introduction to the current version of CIDO, its development, and representative applications.

## Methods

### Coronavirus disease-related data collection

Supplemental Table 1 provides a summary of our coronavirus disease-related data repository, comprising data collected from literature (primarily PubMed and PubMed Central) and from openly available databases. The classifications of viral variants and amino acid variants were obtained from GISAID (https://www.gisaid.org/), NextStrain (https://nextstrain.org/), and WHO. Anti-coronaviral drug information was taken primarily from DrugBank [16] and from data annotated using the Chemical Entities of Biological Interest (ChEBI) ontology [17], COVID-19 diagnostic testing data in this repository are derived from five major sources: (i) the FDA EUA diagnostic testing website (https://www.fda.gov/medical-devices/coronavirus-disease-2019-covid-19-emergency-use-authorizations-medical-devices/in-vitro-diagnostics-euas); (ii) the AdveritasDx database (http://adveritasdx.com/); (iii) the LOINC In Vitro Diagnostic (LIVD) Test Code Mapping for SARS-CoV-2 Tests produced through the collaboration of the FDA, CDC, IICC, Regenstrief Institute, and APHL (https://www.cdc.gov/csels/dls/sars-cov-2-livd-codes.html), and (iv) COVID-19 diagnostic testing kits authorized for use in China (provided by YT). These resources are developed independently and are integrated and are annotated in inconsistent ways. One major task of our work is to use CIDO to support COVID-19 data integration through consistent annotations.

### CIDO ontology development

CIDO development followed OBO Foundry ontology development principles (e.g., openness and collaboration) (4), and utilized the eXtensible Ontology Development (XOD) strategy, which prescribes: ontology term reuse, semantic alignment, use of ontology design patterns for new term generation, and community effort [18]. CIDO’s development started with the reuse and alignment of terms and relations from existing ontologies using the Ontofox tool [19]. We used reference ontologies such as the Ontology for Biomedical Investigations (OBI) [20], Chemical Entities of Biological Interest (ChEBI) [17], Human Disease Ontology (DOID) [21], Human Phenotype Ontology (HP) [22], and Infectious Disease Ontology (IDO) [23] (Supplemental Table 2). CIDO terms are aligned under Basic Formal Ontology (BFO) [24], a top-level ontology conformant to the ISO/IEC 21,838 standard (https://www.iso.org/standard/74572.html). BFO is a domain-neutral framework that has been adopted by more than 450 ontologies as starting point for the creation of terms and definitions in specific domains. It thereby provides a mechanism for overcoming interoperability issues which arise when the attempt is made to integrate ontologies deriving from different sources.

For the generation of terms from domains ranging from amino acid variants to diagnostic medical kits, we developed relevant ontology design patterns and then used the Ontorat tool [25] to automate term generation. For manual term generation and editing, we used the Protégé-OWL editor [26], providing new CIDO specific terms with International Resource Identifiers that start with “CIDO_” followed by 7 automatically generated digits.

We worked closely with ontology development communities to support coronavirus related ontology development. For example, we worked with the Protein Ontology (PR) on generating PR representations of SARS-CoV-2 proteins which were subsequently imported into CIDO. We also periodically submitted issue trackers to other related ontology efforts, for example requests for over 40 specimen-related terms submitted to the Ontology for Biomedical Investigations (OBI) (https://github.com/obi-ontology/obi/issues/1176, also: https://github.com/CIDO-ontology/cido/issues/7). The relevant terms with OBI identifiers and definitions were then imported back into CIDO. Additionally, we have generated many new relations in CIDO to meet our needs, some of which have been proposed for inclusion in the OBO Relation Ontology (RO) [27].

CIDO is designed to support COVID-19 data FAIRness (i.e., findability, accessibility, interoperability, and reusability) [28, 29]. Our ontology development is primarily task-focused and use-case driven. For COVID-19 diagnosis modeling, for example, a team of clinical doctors, diagnosticians, and ontologists, was formed to study COVID-19 diagnosis background [30, 31], collect and annotate available diagnosis kits, focus on specific diagnosis use cases such as [32], design the relevant ontology patterns, and then implement the latter in CIDO.

### CIDO status, source code, deposition, and license

CIDO source code is freely available with the CC-BY license on the GitHub website https://github.com/CIDO-ontology/cido. CIDO has been deposited to the Ontobee ontology repository (http://www.ontobee.org/ontology/CIDO) the BioPortal repository (https://bioportal.bioontology.org/ontologies/CIDO), and the OLS repository (https://www.ebi.ac.uk/ols/ontologies/cido).

### Visual analysis of CIDO by summarization network

The Ontology Abstraction Framework (OAF) tool [33] was used to generate a color image of the layout of the ontology hierarchy (Fig. 1 in Supplemental File 1). To provide a more comprehensible visualization of the most recent version of CIDO, we used the Weighted Aggregate Partial-Area Taxonomy (WAT) summarization network analysis method [34]. By comparing this version with older versions of CIDO we were able to track the evolution of the ontology, as summarized in Supplemental File 1.

**Fig. 1:**
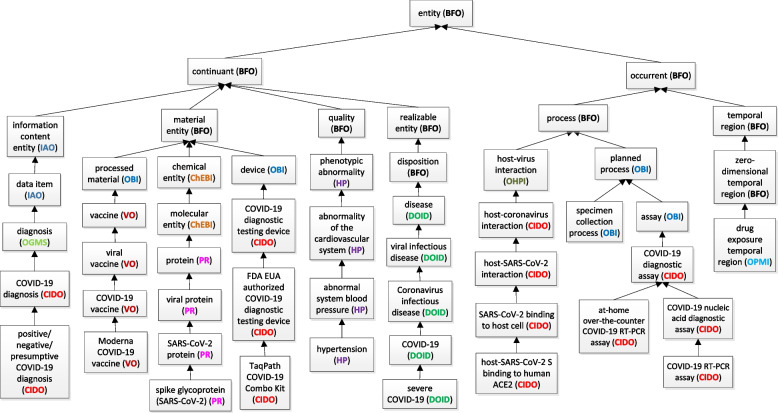
Top level hierarchical structure of class terms represented in CIDO. Abbreviations in parentheses indicate an entity’s source ontology (Supplemental Table 2)

### CIDO applications

In the present communication we describe several applications of CIDO. One use case is the comparative analysis of the shared and different amino acid variants found in the Delta and Omicron variants, with the purpose of better understanding the mechanisms of coronavirus evolution, transmission, and virulence. Another use case is a SARS-CoV-2 drug repurposing study. Using the knowledge represented and classified in CIDO, we systematically queried the host-coronavirus protein-protein interactions, anti-coronavirus drugs, and protein targets of different drugs, with the goal of identifying and designing possible drugs with a potential for optimized treatment performance.

## Results

### The upper level structure and design pattern of CIDO

Figure 1 lays out the high-level hierarchical structure of CIDO and shows the various imported external ontologies. Areas related to the coronavirus infectious disease represented by CIDO include: coronavirus taxonomy, coronavirus variants, genes and proteins and their mutations, phenotypes, diseases, epidemiology, diagnosis, host-coronavirus protein-protein interactions, vaccines, and drugs. All the terms are aligned under the top-level Basic Formal Ontology (BFO) (7) (Fig. 1). CIDO imports terms from over 20 reference ontologies from the OBO ontology library, with the representative ontologies introduced in Supplemental Table 2 and Fig. 1.

In addition to importing terms from existing ontologies, we have also generated many CIDO-specific terms e.g., resources for SARS-CoV-2 viral variants, amino acid mutations, and diagnostic medical device kits. New axioms, such as those linking different types of proteins and other molecules that are related to host-coronavirus protein-protein interactions (PPIs) and drug-target interactions, have also been developed for CIDO. In the version released on August 1, 2022, there are 370 relations used in CIDO, including 87 relations newly generated with “CIDO_” prefix. Admittedly, some of the newly generated relations in CIDO may be more suitable for the more general level Relation Ontology (RO) [27]; future research will involve further refinement of these relations.

Our previous *Comment* paper in *Scientific Data* [12] describes the general CIDO design pattern that lays out the relationships among selected major entities modeled in the ontology. In the next sections, we provide details of specific ontological modeling and representation provided in CIDO.

### Ontological classification of coronaviruses and coronavirus variants

CIDO imports resources from the NCBITaxon to represent various coronaviruses and their relations [13]. SARS-CoV and SARS-CoV-2 belong to the Sarbecovirus, a subgenus of the genus Betacoronavirus. MERS-CoV belongs to Merbecovirus, a sibling to Sarbecovirus. Four human coronavirus strains (229E, NL63, HKU1, and OC43) cause mild common colds in humans, where 229E and NL63 belong to Alphacoronavirus, and HKU1 and OC43 belong to Embecovirus under Betacoronavirus.

We have generated 39 CIDO specific classes to represent specific COVID-19 viral variants. CIDO defines distinct viral variants of SARS-CoV-2 based on 3 classification methods: GISAID clades [35], PANGO lineages [36], and WHO clades [https://www.who.int/en/activities/tracking-SARS-CoV-2-variants/]. A viral variant is defined as a virus that has undergone variation such that there is a characteristic set of mutations in comparison to the reference virus sequence. These variants include various genetic mutations resulting in changes in transmission, infectivity, and virulence as compared to the original Wuhan reference strain. The GISAID clades and PANGO lineages both utilize the same data set but utilize different clustering algorithms to designate specific variants. PANGO lineages also differ by defining characteristic mutations that occur in a majority of specific SARS-Cov-2 variants while GISAID variants define universal mutations. The following examples illustrate these three hierarchies:

*‘SARS-CoV-2 Delta virus’: ‘is a’ some ‘SARS-CoV-2 based on WHO classification’*

*‘SARS-CoV-2 BA.5 virus’ ‘is a’ some ‘SARS-CoV-2 based on PANGO lineage’*

*‘SARS-CoV-2 clade G virus’: ‘is a’ some ‘SARS-CoV-2 based on GISAID clades’*

WHO utilizes GISAID clade and PANGO lineage representations as synonyms for epidemiologically relevant variants, designated either as a Variant of Concern (VoC) or as a Variant of Interest (VoI) [15]. VoIs are variants that are identified as having the potential to become VoCs through causing increased transmission or worse disease processes. VoCs remain designated as such until they are no longer prevalent.

### Ontological representation of SARS-CoV-2 proteins and genes

**Fig. 2:**
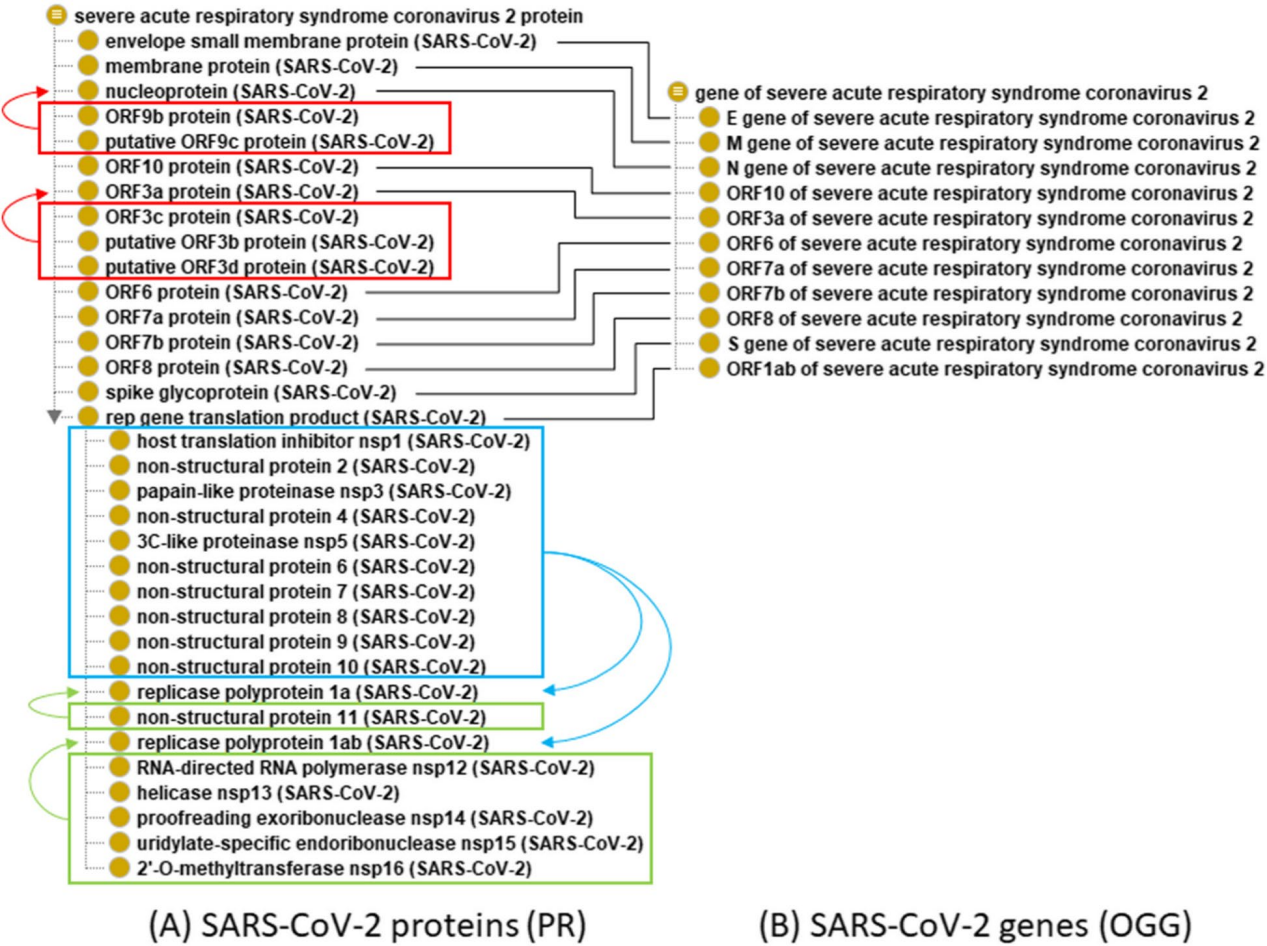
SARS-CoV-2 proteins and genes. **A** PR modeling of SARS-CoV-2 proteins. **B** OGG modeling of SARS-CoV-2 genes. Black lines represent the ‘has gene template’ relation connecting proteins to genes. Red boxes denote proteins translated from ORFs that are internal to or overlap with those of the longer indicated gene (red arrows). The light blue box indicates proteins that are produced by proteolytic processing of either replicase polyprotein 1a or replicase polyprotein 1ab, while green boxes indicate those that derive specifically and uniquely from pp1a or pp1ab

CIDO imports terms for SARS-CoV-2 proteins from the Protein Ontology (PR) and terms for SARS-CoV-2 genes from the Ontology of Genes and Genomes (OGG), a simplified representation of which is shown in Fig. 2. Gene terms are based on those found in the NCBI Gene database [37] while proteins are as given by UniProtKB [38] [https://www.uniprot.org/uniprot/?query=proteome:up000464024], with cross-reference information from NCBI RefSeq [https://www.ncbi.nlm.nih.gov/protein?term=(sars-cov-2%20Wuhan-Hu-1%20AND%20refseq%5Bfilter%5D)]. CIDO represents only those genes that are described in NCBI Gene, and only those proteins (and their derivatives) that are described in UniProtKB. There are other protein open reading frames (ORFs) such as ORF2b (aka S.iORF1) [39], ORF-Sh and ORF-Mh [40], which are held in reserve, but they will be added should they gain experimental or database support. A full comparison between PR, RefSeq, and UniProtKB is given in Supplemental Table 3 with respect to accessions, genes, and names used (protein length and evidence for existence are also presented).

In general, PR uses SARS-CoV-2 protein names as given in UniProtKB and gene names as given in RefSeq, wherever these are available. A key difference between the PR representation and those of RefSeq and UniProtKB is that the former has a single record for each proteolytic cleavage product of the ORF1ab (aka rep) gene, while each of the latter resources has two records for the subset of products that are encoded by both the polyprotein 1a (pp1a, aka ORF1a) and the polyprotein 1ab (pp1ab, aka ORF1ab) transcript (where the latter is the result of -1 ribosomal frameshifting). Both polyproteins are further processed by proteolytic cleavage; processing of either will yield ten identical chains (Fig. 2A, light blue box), while one additional chain is unique to ORF1a and five additional chains are unique to ORF1ab (green boxes). In addition, PR unites each of the polyproteins under the grouping term ‘rep gene translation product’ (the synonym is used here to prevent confusion with the ORF1ab transcript-derived polyprotein). Several proteins are translated from alternative ORFs within or overlapping transcripts that also produce longer proteins (red boxes). One of these, ORF9b, has been demonstrated (in SARS-CoV-1) to use leaky ribosome scanning [41]; potentially this mechanism applies to the others as well, though the existence of the ORFs labeled ‘putative’ is questionable [42]. All SARS-CoV-2 proteins are grouped under ‘severe acute respiratory syndrome coronavirus 2 protein’. In total—not counting the grouping terms—there are forty SARS-CoV-2-related PR terms. Currently, none of these represent proteoforms with amino acid modifications; these will be added in the future.

### Ontological representation of SARS-CoV-2 amino acid variants

In addition to the representation of viral variants, CIDO also defines and represents various amino acid (AA) variants. Similar to the viral variant definition, an AA variant is defined in CIDO as “An amino acid in a protein that varies from another amino acid in comparison to the reference protein”. CIDO further defines the object property *‘is characteristic AA variant’* to describe a relation between an AA variant and a protein where the AA variant is a characteristic AA variant of a specific viral variant. An AA variant is defined as characteristic when the presence of the AA can be used to identify the AA variant. We characterize these variants by comparing the amino acid at a given position to the reference wild-type strain. For example, the D614G mutation in the spike polyprotein (S:D614G) is well known for emerging in several VoCs and has been proven to increase SARS-CoV-2 infectivity [43]. The CIDO class ‘D-614G in SARS-CoV-2 S protein’ (where S protein is just as the spike protein) has the following axioms (Fig. 2):

*‘D-614G in SARS-CoV-2 S protein’:*

- *‘characteristic AA variant of’ some ‘SARS-CoV-2 Omicron variant’*
- *‘is a’ some ‘AA variant in SARS-CoV-2 S protein S1 RBD region’*
- *‘has amino acid position’ value 614*
- *‘has part’ some ‘glycine residue’*
- *‘has mutated from’ some ‘aspartic acid’*

However, the above framework does not work well for describing characteristic deletions or other mutation events. As the amino acid that was deleted does not exist, this leads to issues where the ontology asserts that something holds of ‘all coronaviral amino acids’. To address this issue, we define the AA deletion as a process. Moreover, this variation process can be generalized to include any mutation event. The relationship between the deletion process and a resulting AA variant, is defined as:

*‘A888- deletion in SARS-CoV-2 S protein’: ‘is AA mutation of’ some ‘SARS-CoV-2 S protein’*

as shown in Fig. 3.

**Fig. 3:**
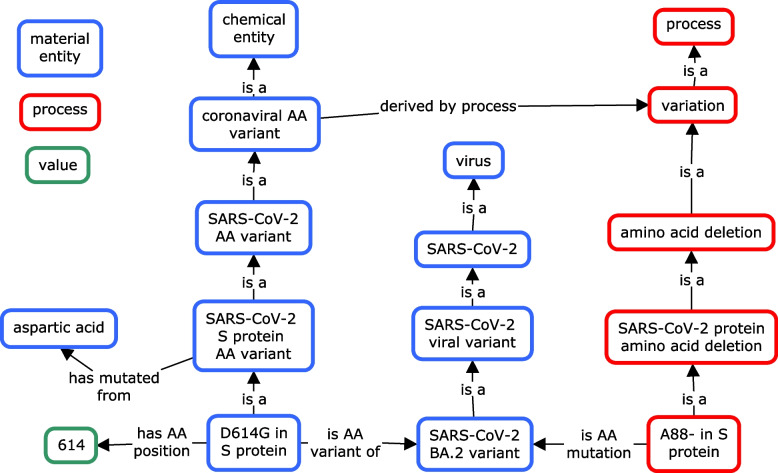
CIDO modeling of AA variants and mutations. CIDO represents AA variants as material entities if they are substitutions and AA mutations as processes to represent deletions in SARS-CoV-2 microbial variants. Both AA variants utilized analogous axioms due to differences in continuants and occurrents

### Host phenotype modeling in CIDO

CIDO contains terms for 18 symptoms and 22 comorbidities commonly found in COVID-19 patients [44]. These symptoms and comorbidities are mapped to phenotypes in the Human Phenotype Ontology (HP) from where they are imported back into CIDO. To link these symptoms and comorbidities as they occur in relation to COVID-19, we have also generated new relations *‘disease susceptibly has phenotype’* and *‘disease susceptibly severe with comorbidity’*. The first relation represents the relation between a disease process and a phenotype where the person with the disease is susceptible to having that phenotype. The second is a shortcut relation between a disease process which is susceptible to becoming more severe when the patient has the comorbidity. Examples of usage of these relations are:

- *SARS-CoV-2 disease process: ‘disease susceptibly has phenotype’ some Fever.*
- *SARS-CoV-2 disease process: ‘disease susceptibly severe with comorbidity’ some hypertension.*

CIDO also represents the relation between SARS-CoV-2 variant and specific phenotypes, for example, the relation between the Delta variant and the formation of syncytia in lungs [45]:

*‘Delta variant disease process’: ‘bearer of disease susceptible to phenotype’ some syncytia*

We are in the process of evaluating and submitting some of our newly generated relations to the OBO Relation Ontology (RO) as they may be more appropriate for inclusion there. For example, we have submitted two new relation terms *‘evolves into’* and *‘evolves from’* to the RO issue tracker (https://github.com/oborel/obo-relations/issues/620). If these relations are added to RO, we will then obsolete our original CIDO relation terms and replace them with the new RO terms.

### Ontological modeling of epidemiology and public health

CIDO includes many terms related to the epidemiology of COVID-19, derived primarily from the Infectious Disease Ontology (IDO) [23] and the Virus Infectious Disease Ontology (VIDO) [14]. Recent research [46, 47] highlights the importance of viral load to SARS-CoV-2 transmission rates. Indeed, Wuhan, Delta, and Omicron strains are associated with distinct peak viral loads with respect to different demographics. VIDO characterizes ‘viral load’ as the proportion of virions to volume of a given portion of fluid in which the virions are located. VIDO provides a datatype property ‘has viral load measurement’ which supports representation of viral load values. For example, an instance of OBI’s class blood plasma specimen from an instance of a host infected by SARS-CoV-2 can be (partially) represented as having a viral load value in the following manner:

‘*blood plasma specimen 1’ rdf:type ‘blood plasma specimen’*

*and ‘has part’ some ‘SARS-CoV-2’*

*and ‘has viral load measurement’ value 10*^*8*^

Additionally, VIDO provides virus-specific terminological content that can be extended in CIDO to represent other important epidemiological terms, such as *COVID-19 prevalence*, *SARS-CoV-2 infectivity*, and *COVID-19 mortality rate*.

Moreover, CIDO includes resources needed for comparison of transmission differences among SARS-CoV-2 variants. The Omicron variant is significantly more transmissible than the reference Wuhan strain and Delta strain. The transmission rate is often represented using R0, the basic reproduction number that measures the transmissibility of infectious agents [48]. The average R0 values for the Wuhan reference strain, Delta strain, and Omicron BA.1 strain are 2.69 [49], 5.02 [50], and 9.05 [51], respectively. Accordingly, we have generated a data property relation ‘has average R0’, which can be used to represent the R01 value of each variant:

*‘SARS-CoV-2 reference strain: ‘has average R0’ value 2.69*

*‘SARS-CoV-2 Delta variant’: ‘has average R0’ value 5.02*

*‘SARS-CoV-2 Omicron BA.1 variant’: ‘has average R0’ value 9.05*

### COVID-19 diagnosis testing modeling in CIDO

During a pandemic, the availability of fast and accurate diagnostic testing is essential to control the situation. Because SARS-COV-2 is a novel virus, the traditional pathway to approve a testing kit to be used in the market will not satisfy the urgent demand in a timely manner. In the US, an Emergency Use Authorization (EUA) under Section 564 of the Federal Food, Drug, and Cosmetic Act (FD&C Act) allows the special authorization and use of drugs and other medical products during emerging infectious disease threats such as the COVID-19 pandemic. From 2020 March until now, the US Food and Drug Administration (FDA) has authorized hundreds of different types of in vitro diagnostic tests under the EUA authorizations. To make those EUA diagnostic testing data Findable, Accessible, Interoperable, and Reusable (FAIR) [28], it is important that the testing kits used are registered in a structured and machine-readable manner.

CIDO comprises representations of 345 molecular and serological diagnostic tests authorized by the FDA. We created a term ‘*COVID-19 diagnostic testing device*’ and its child term ‘*FDA EUA authorized COVID-19 diagnostic testing device*’, where the latter is to be the home of all FDA EUA authorized In Vitro Diagnostics (IVD) tests for COVID-19.

**Fig. 4:**
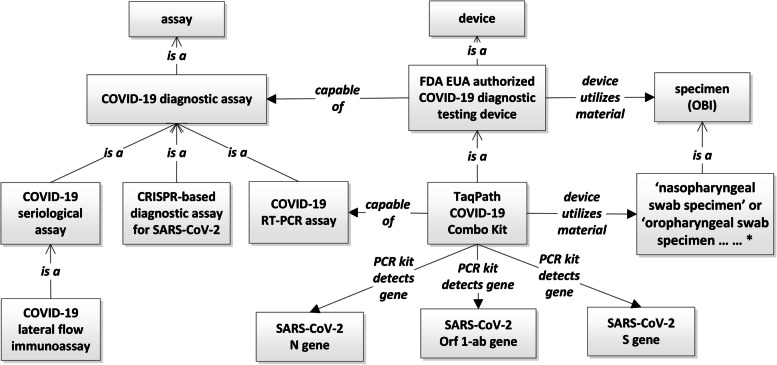
Modeling of COVID-19 diagnostic testing using CIDO. *, only two out of six specimen terms are shown in this figure

An example representation of the TaqPath COVID-19 Combo Kit from Thermo Fisher Scientific, Inc., which was authorized under an EUA authorization (https://www.fda.gov/media/136113/download) is shown in Fig. 4, which lays out the current CIDO representation of device, assay, diagnostic process and genes that the test is designed to detect. A device ‘*TaqPath COVID-19 Combo Kit’* is *‘capable of’* a *‘COVID-19 RT-PCR assay’*. This test detects the existence of N, S and ORF-1ab gene regions that are part of the corresponding genes of the SARS-CoV-2 reference strain. We created a short-cut relation *‘PCR kit detects gene’* to represent a direct relationship between a diagnostic testing kit and the target gene/sequence fragments. Another short-cut relation ‘*device utilizes material*’ was created to link the diagnostic testing and the tested specimen. This relation can be logically represented as a property chain (https://github.com/oborel/obo-relations/issues/497):

This particular diagnostic testing kit can utilize 6 specimen types, as again shown in Fig. 4. The following axiom represent the ontological arrangement of such a relation using a union of 6 specimen terms:

*‘device utilizes material’ some (‘nasopharyngeal swab specimen’ or ‘oropharyngeal swab specimen’ or ‘anterior nasal swab specimen’ or ‘mid-turbinate nasal swab specimen’ or ‘nasopharyngeal aspirate specimen’ or ‘bronchial alveolar lavage’)*

Using the strategy defined here, we systematically collected and used CIDO to model and represent over 300 molecular and serological diagnostic tests, including 225 SARS-CoV-2 RT-PCR assays, authorized by US FDA. All the 343 tests are annotated with a total of ten COVID-19 diagnostic technologies, such as RT-PCR, LAMP, Next Generation Sequencing, a CRISP-based method, ELISA, lateral flow immunoassay, chemiluminescent, and so on.

### CIDO modeling and representation of host-coronavirus protein*-protein interactions and drugs*

CIDO represents over 300 experimentally verified host-coronavirus protein-protein interactions (PPIs), over 300 anti-coronaviral chemicals and/or their corresponding drugs, and over 400 drug targets. Here the coronaviral proteins may derive from SARS-CoV, MERS-CoV, or SARS-CoV-2. In early 2020, we performed literature mining and identified 110 chemical drugs and 26 antibodies effective, either in vitro or in vivo, against at least one human coronavirus infection, where the human coronaviruses involved are primarily SARS-CoV and MERS-CoV [52]. Our ontological representation, classification, and analysis of these drugs yielded many potentially valuable scientific insights. Since early 2020, we have collected more drugs and chemicals with a focus on those against SARS-CoV-2. Furthermore, we have collected and annotated representations of further PPIs and chemical-drug interactions.

All CIDO-represented host-coronavirus PPIs are experimentally verified and reported in the literature. For example, CIDO has recorded 332 physically associated PPIs identified by the affinity-purification mass spectrometry assay [5]. These PPIs involve both proteins from the SARS-CoV-2 side and the host side, and many of these coronaviral and host proteins are also targets of multiple drugs.

In CIDO, each host-coronavirus PPI is defined to have at least two participants, including one protein from a coronavirus and one from its host. For example, the ‘host-SARS-CoV-2 protein-protein interaction’ is defined as:

*(‘has participant’ some ‘SARS-CoV-2 protein’) and (‘has participant’ some (organism and ‘has role’ some ‘host role’))*

**Fig. 5:**
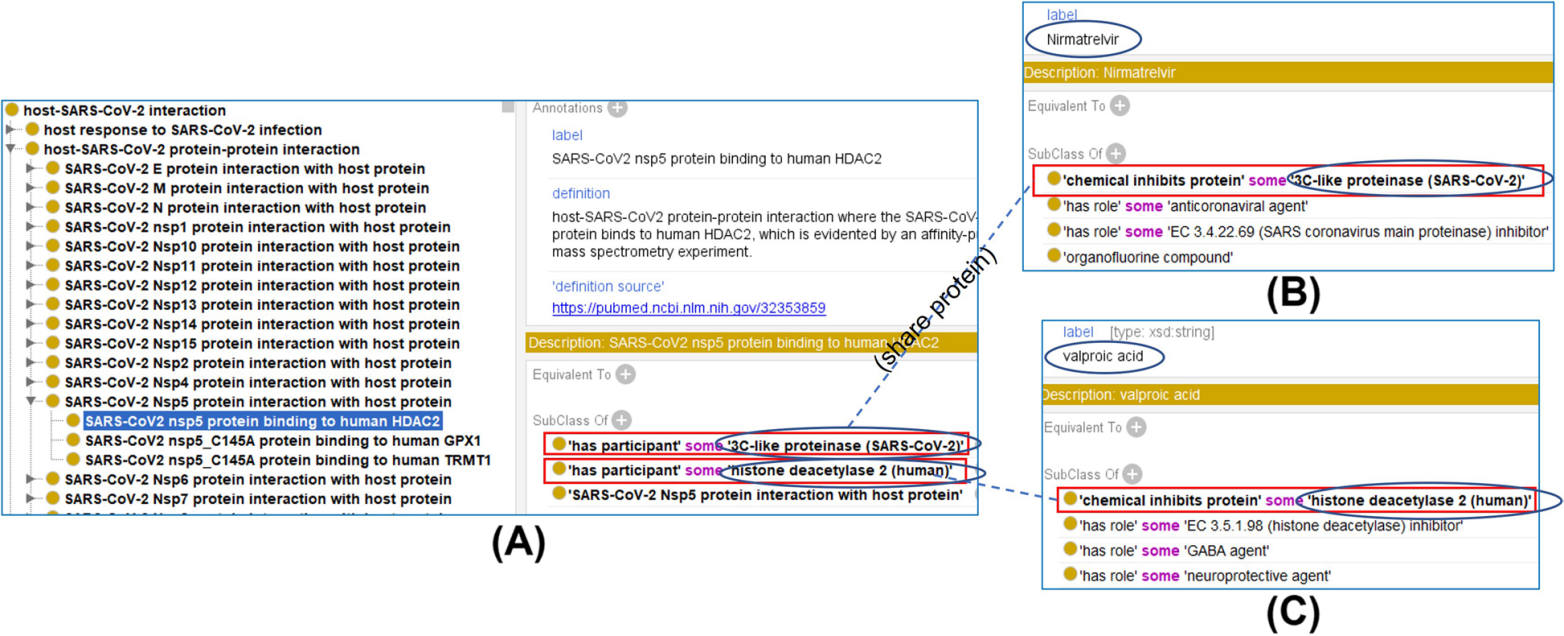
Host-coronavirus protein-protein interactions (PPIs) and drugs targeting the viral or host proteins. **A** The hierarchy of PPIs, including ‘SARS-CoV-2 nsp5 protein binding to human HDAC2’. **B** The chemical nirmatrelvir (a component of the Pfizer drug Paxlovid) is an inhibitor of the virus protein nsp5 (i.e., 3C-like proteinase), which is critical for viral replication. **C** A chemical ‘Valproic Acid’ is an inhibitor of the HDAC2 (i.e., histone deacetylase 2). Valproic acid is also a valuable candidate against SARS-CoV-2

Figure 5 illustrates how CIDO represents hundreds of host-SARS-CoV-2 PPIs, drug active ingredients, and chemical-protein interactions. Specifically, there are three specific PPIs under the class ‘SARS-CoV-2 nsp5 protein interaction with host protein’, such as ‘SARS-CoV-2 nsp5 protein binding to human HDAC2’. This example PPI has two participants:

*‘has participant’ some ‘3C-like proteinase (SARS-CoV-2)’*

*‘has participant’ some ‘histone deacetylase 2 (human)’*

Note that 3C-like proteinase, another name for nsp5, can be inhibited by the chemical nirmatrelvir, a component of the Pfizer drug Paxlovid. Human histone deacetylase 2 (i.e., HDAC2), can be inhibited by a chemical ‘Valproic Acid’, which has been found valuable against SARS-CoV-2 [53]. These relations are logically defined in CIDO as follows (Fig. 5B and C):

*'nirmatrelvir': ‘chemical inhibits protein’ some ‘3C-like proteinase (SARS-CoV-2)’*

*‘Valproic Acid’: ‘chemical inhibits protein’ some ‘histone deacetylase 2 (human)’*

### Anti-coronavirus vaccine representation in CIDO

As the developers of the Vaccine Ontology (VO) [54], we (YH, AL, AH, PH) first represented a total of over 100 COVID-19 vaccines at different stages (licensed, authorized, in clinical trials, or verified with laboratory animal models) in VO, and then imported these terms from VO to CIDO (Fig. 1, Supplemental Table 2). In total, we have imported over 300 terms from the VO to CIDO. Furthermore, we have developed Cov19VaxKB, a web-based Integrative COVID-19 vaccine knowledge base, which has used ontologies including the VO to represent, classify, and analyze various COVID-19 vaccines and vaccine components (e.g., vaccine adjuvants), and vaccine adverse events [55]. We have also developed reverse vaccinology and machine learning methods to predict vaccine antigen candidates [56]. The functions and immune mechanisms of these candidates are being further analyzed using ontology-based approaches [15]. Furthermore, we have been using CIDO and other ontologies including the Ontology of Adverse Events (OAE) to systematically examine adverse events associated with SARS/MERS/COVID-19 vaccine candidates.

### Clinical metadata type representation in CIDO

To support classification and analysis of clinical data, CIDO includes representations of many clinical metadata types. Metadata is the data that provides information about other data. In our study of COVID-19 related clinical data, we have focused on two use cases: the analysis of vaccine adverse events using the VAERS data resource as described above and the analysis of the clinical data from the National COVID Cohort Collaborative (N3C) program [57]. The N3C system is a collection of harmonized clinical data on COVID-19 from contributing data partners. N3C data is represented using the OMOP common data model (CDM). From the OBO ontology point of view, OMOP has its issues such as the lack of semantics, ambiguities, and hidden assumptions [58]. In our N3C related clinical data study, we have focused on the mapping of the OMOP CDM elements and OBO ontologies and adding semantic relations among terms.

Table 1 lists the representative clinical metadata types that are primarily mapped to the OMOP CDM elements. These are general clinical data types applicable to studies not only of COVID-19 but also of other human diseases. As a result, all these terms are imported from other reference OBO ontologies. The Ontology of Precision Medicine and Investigation (OPMI) [59, 60], another OBO library ontology, has been used as a major reference ontology to represent those clinical data types not found in other OBO ontologies (Table 1). After the mapping of OMOP CDM elements to OBO ontologies, we imported these mapped terms to CIDO to support COVID-19 clinical data annotation and analysis.

**Table 1:** Representative clinical metadata types covered in CIDO. All listed examples are considered classes in the ontology

Metadata types	Metadata Examples
person (NCBITaxon_9606)	person ID (OPMI_0000470), gender (PATO_0001894), year of birth (OPMI_0000473), race (NCIT_C17049), ethnicity (NCIT_C16564), care site (OPMI_0000479), geographic location (GAZ_00000448)
specimen (OBI_0100051)	specimen ID (OBI_0001616), date of specimen collection (OBIB_0000714), anatomical structure (UBERON_0000061)
visit occurrence (OPMI_0000482)	visit occurrence identifier (OPMI_0000483), visit start date (OPMI_0000487), visit end date (OPMI_0000488), preceding visit occurrence (OPMI_0000492), ER visit (OPMI_0000486)
procedure occurrence (OPMI_0000505)	procedure (NCIT_C25218), procedure start date (OPMI_0000508), procedure end date (OPMI_0000510), care provider (OPMI_0000163)
drug exposure (OPMI_0000572) and device exposure (OPMI_0000554)	drug (CIDO_0000167), drug exposure start time (OPMI_0000565), drug exposure end time (OPMI_0000567), medical device (NCIT_C16830), diagnostic kit (CIDO_0000453)
clinical measurement (CMO_0000000)	clinical measurement identifier (OPMI_0000582), care provider (OPMI_0000163), measurement time (OPMI_0000579), measurement unit label (IAO_0000003), measurement date (OPMI_0000580)
observation period (OPMI_0000575)	observation period start date (OPMI_0000577), observation period end date (OPMI_0000578), provenance of observation record (OPMI_0000522)

In the OMOP / N3C data structure, each concept set groups terms into what are called value sets. A value set is a set of codes selected from those defined by one or more code systems to specify which codes can be used in a particular context. However, their grouping is heuristic and not ontology-based. The ontology support is an ongoing project. OMOP2OBO is the first health system-wide integration and alignment system that systematically maps over 23,000 concepts from OMOP standard clinical terminologies to OBO concepts [61]. While OMOP2OBO is more focused on the value set mapping, our mapping and further term generation (Table 1) is more focused on the small set of the core OMOP CDM concept set meta elements. The two complementary systems can be used together to support robust clinical COVID-19 data annotation, integration, and analysis.

### Visual evolution analysis of CIDO

**Fig. 6:**
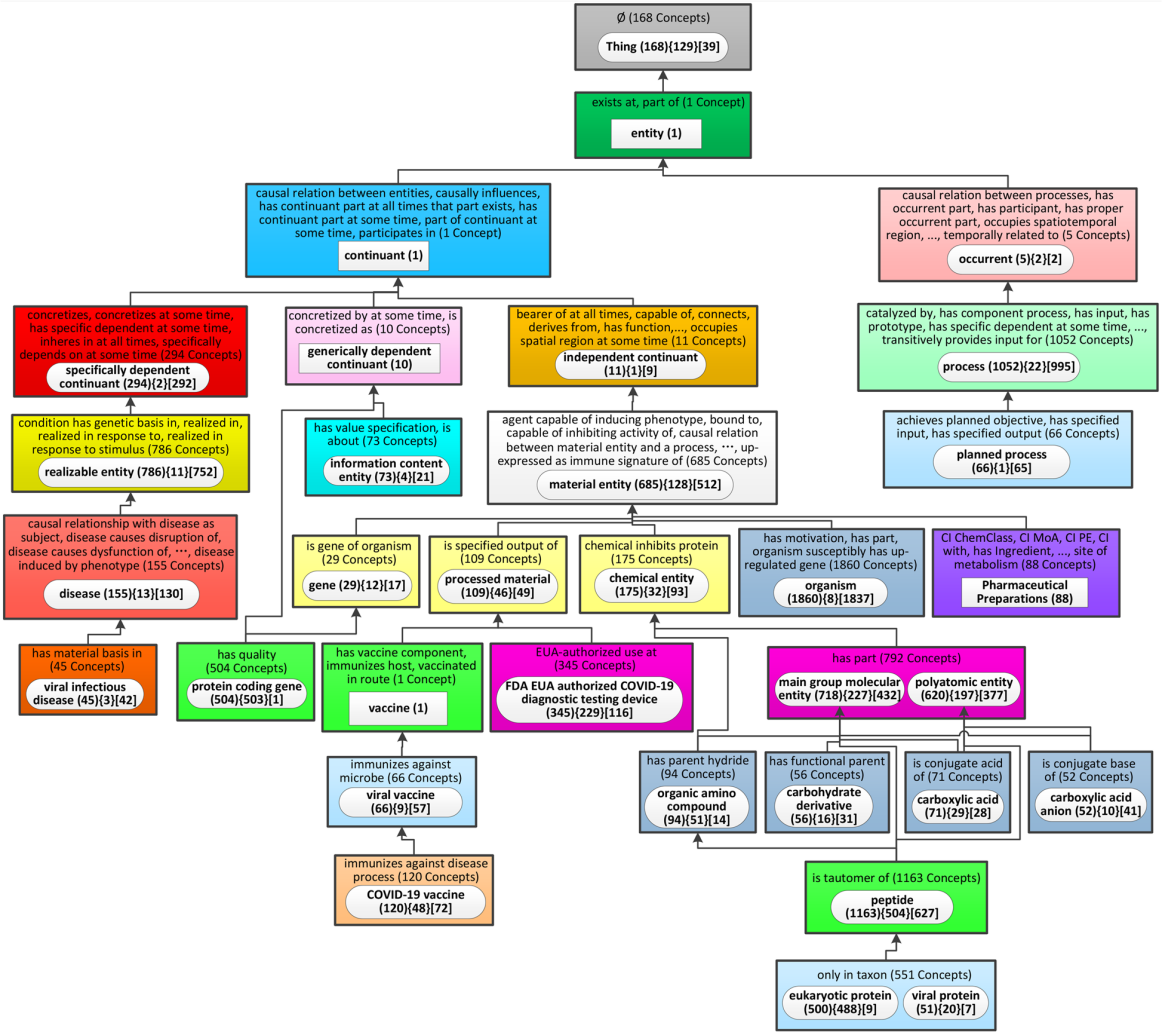
The weighted aggregate taxonomy (WAT) for CIDO (version 1.0.306) with 10,853 concepts (b = 42). A white node inside a colored rectangular box represents a partial-area, which is a group of concepts having the same set of nonhierarchical (lateral) relationships and similar semantics denoted by the concept listed inside the white node. Relationships are listed inside the colored box (inherited ones are not shown). The boxes are color-coded by cardinalities of their sets of lateral relationships. Upward arrows are the hierarchical relationships connecting partial-areas. The weight of a partial-area is defined as the number of descendant concepts. A partial-area with a weight less than b is small and is aggregated into its closest ancestor large partial-area. A large partial-area having no aggregated partial-areas is represented as a rectangle white box with one number indicating the number of summarized concepts. A large partial-area having aggregated partial-areas is represented as a rectangle with rounded corners and with three numbers. The first number inside () is the number of summarized concepts including concepts aggregated from small partial-areas, the second number inside {} is the number of small partial-areas aggregated into it, and the third number inside [] is the number of concepts of the partial-area before the aggregation**.** See more details in Supplemental File 1

To provide a condensed and comprehensive visualization of CIDO, we have previously developed a new Weighted Aggregate Partial-Area Taxonomy (WAT) summarization network method and used it to analyze an early version (version 1.0.108) of CIDO with a total of 5138 concepts [34]. Since then, newer versions of CIDO that include more concepts have been generated. To evaluate these new additions to CIDO, we have generated a new WAT summarization network that visualizes CIDO version 1.0.306 with 10,853 concepts (Fig. 6). As shown in Fig. 6, major branches of CIDO include infectious diseases, genes, vaccines, chemicals, and COVID-19 testing devices.

Comparing the old version (Fig. 2 in Supplemental File 1) with the new, we can identify which nodes had a considerable increase in the number of new descendant terms. For example, “COVID-19 vaccine” (120){48} [72] has been added to the ontology visualization (Fig. 6). The number (120) means that the term “COVID-19 vaccine” includes 120 descendant terms, with 48 of those aggregated from 48 descendant nodes of “COVID-19 vaccine,” each of which has only one term (less than b = 42), and 72 representing all other descendant terms of the large partial-area “COVID-19 vaccine” before the aggregation. By expanding this node in the manner supported by the OAF tool, we can see some interesting newly added vaccine terms such as “Pfizer–BioNTech COVID-19 vaccine”, “Moderna COVID-19 vaccine”, “Oxford–AstraZeneca COVID-19 vaccine”, and “Nanocovax”. In contrast, the old version includes only one term for “COVID-19 vaccine” without any descendant term. Another example is “FDA EUA authorized COVID-19 diagnostic testing device” (345){229}[116] in Fig. 6 including terms “COVID-19 Nucleic Acid RT-PCR Test Kit” and “BinaxNOWTM COVID-19 Ag Card Home Test” for which there are no corresponding terms in the old version.

#### Use cases of CIDO

CIDO has been proposed and used in many applications by us or the wider community as exemplified by references [15, 44, 52, 62–67]. Five use cases of our own application of CIDO are introduced here.

1. Ontology-based coronavirus-related knowledge and data standardization, annotation, mapping, integration, and inferencing, supporting advanced COVID-19 data analysis

As a reference ontology in the field of coronavirus infectious disease, CIDO provides a standard representation and definitions of terms and axioms in various areas related to COVID-19 and other coronavirus diseases. The above sections have provided details on how CIDO standardizes and classifies terms and relations in different domains related to coronavirus diseases. Usage of the CIDO standard representation enhances data FAIRness, annotation, and integration.

The COVoc Controlled Vocabulary for COVID-19 is an application ontology developed by the European Bioinformatics Institute (EMBL-EBI) and the Swiss Institute of Bioinformatics (SIB) in March 2020 [14]. The primary usage of COVoc is to enable seamless annotation of biomedical literature to core databases and tools at ELIXIR (a European-wide intergovernmental organization for life sciences). COVoc utilizes existing OBO ontologies and other vocabularies to augment connections to other useful resources such as the COVID-19 Data Portal (https://www.covid19dataportal.org/), as well as assisting in the curation and annotation of COVID-19 literature. CIDO has been working with COVoc to ontologize many terms in COVoc for better COVID-19 data annotations.

In addition to the USA and Europe, CIDO has also been applied in many other countries including China. CIDO has also been recommended as one of the semantic standards in areas related to clinical data integration and annotations by the National Population Health Data Center in China (NPHDC). It is included in their population health data archive (PHDA) [68] and provides ontology services in MedPortal [69]. And it has been also used for the construction of knowledge graphs about COVID-19 [70].

Since CIDO incorporates multiple different types of knowledge about coronavirus diseases, it can be used both to query and infer new scientific insights and to reason from analysis of clinical data. This reasoning is enabled by the structure of the knowledge base used by CIDO. CIDO provides a T-box vocabulary, i.e., a general terminological constraints for representing COVID-19 phenomena. CIDO’s vocabulary can then be used to generate new data once instance-level data, the set of which in the knowledge base is called the A-box, has been ingested into the knowledge base. Data organized by CIDO is multiplied in value through the inferences enabled by the ontological axioms included within it.

An example in our ontology-based clinical COVID-19 data analysis is our analysis of differential COVID-19 symptoms during the early pandemic [44]. In this study, we classified different symptom phenotypes in relation to pandemic locations, time periods, and comorbidities. The 18 most common COVID-19 symptoms were mapped to the HPO terms and imported to CIDO. Based on the HPO classification, we grouped these symptoms into further categories. For example, we grouped 4 COVID-19 related symptoms (nausea, vomiting, abdominal pain, and diarrhea) under *abdominal system symptoms*, and we grouped three symptoms (headache, loss of smell, and loss of taste) under *nervous system symptoms*. In addition, CIDO provides semantic representation of knowledge learned from clinical data analysis. An example is our representation of how symptoms and comorbidities are linked to COVID-19 disease [44]. Note that we emphasize the use of ‘susceptibility’ (a subclass of ‘disposition’) to represent this knowledge, for example when dealing with clinical phenotypes, vaccine/drug adverse events, and immune deficiency association.

Another use case is the CIDO modeling of the molecular mechanisms of acute kidney injury (AKI) [71]. AKI is a commonly found phenotype among hospitalized COVID-19 patients. Our extensive literature mining and analysis of the BioGRID COVID-19 interaction data identified 3 key physiological processes (i.e., RAS activation, complement activation, and systemic inflammation) and many interactors like CD147, CD209, CypA, and MASP2 that are heavily implicated in these processes. CIDO was used to represent our analyzed results, leading to further understanding of the COVID-19 associated AKI mechanisms [71, 72].

(2) CIDO queries for Delta and Omicron differences for better mechanistic understanding of virulence and transmission

**Fig. 7:**
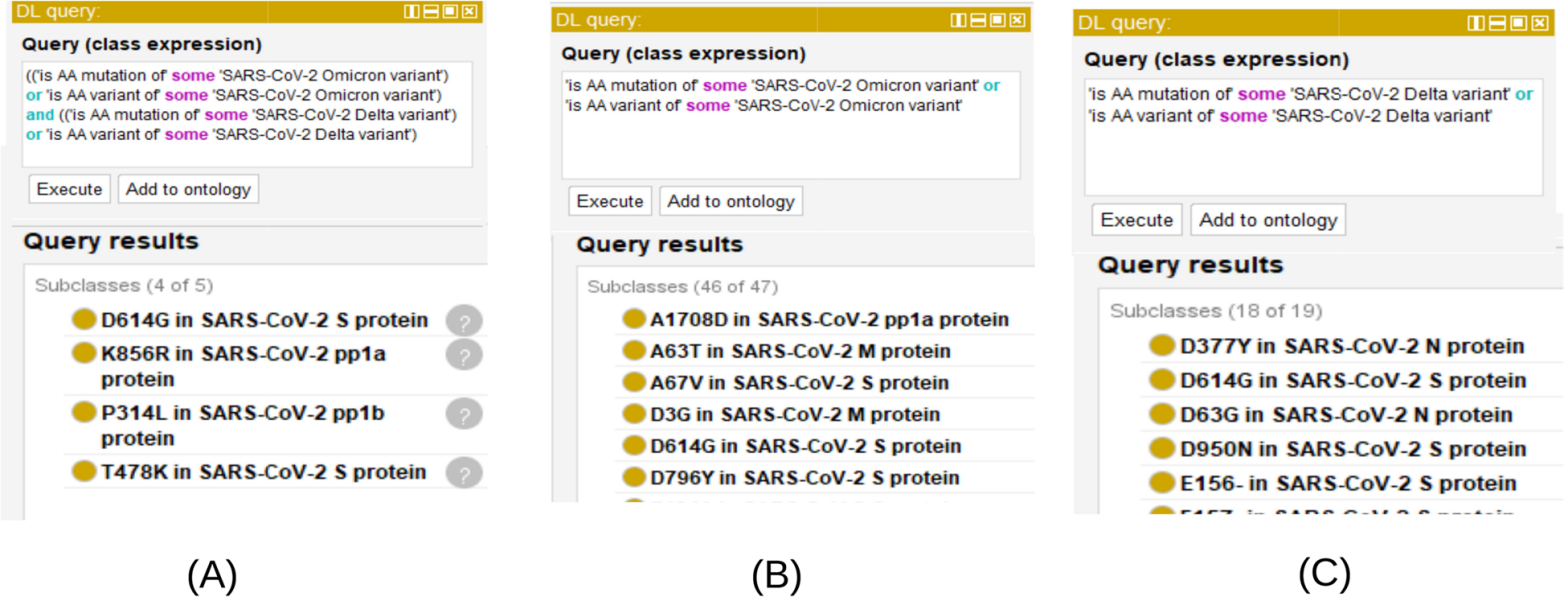
Query CIDO amino acid (AA) variants for Delta and Omicron strain comparison and basic transmission and virulence mechanism understanding. **A** DL query for AA variants shared by Delta and Omicron strains. **B** DL query for amino acid variants that belong to Omicron. **C** DL query for amino acid variants that belong to Delta. Current AA variants for Omicron and Delta strains are also characteristic AA variants

Among many SARS-CoV-2 variants, the Omicron strain is more transmissible but less virulent than the Delta strain, and both strains are more transmissible than the Wuhan reference strain [73–75]. We hypothesized that these differences reflect underlying differences in amino acid (AA) variants. CIDO includes 92 specific CIDO terms representing characteristic mutations and 35 further mutations that are not considered as characteristic. CIDO allows for easy comparison of coronaviral AA variants that are associated with specific SARS-CoV-2 variants. To address the above hypothesis, we can perform specific queries to compare the AA variants in the two strains with the aim of uncovering the molecular mechanisms underlying the different phenotypes (Fig. 7).

Figure 7A shows a DL query that searches CIDO for the characteristic amino acid variants shared between SARS-CoV-2 Delta strain and Omicron strain. The results show four such variants: D614G and T478K in S protein, K856R in pp1a [nsp3] protein, and P314L in pp1b [nsp12] protein. S:D614G increases infectivity by allowing for a greater binding ratio of the S-protein trimer units to hACE2 [76]. T487K has similarly shown to increase the actual binding affinity to SARS-Cov-2 [77]. While the specific effects of K856R and P314L are unknown, both mutations are located in proteins responsible for viral replication [78, 79]. K856R is located in the region responsible for cleaving the non-structural proteins from pp1ab [78]. P314L however, is part of the RNA polymerase which is responsible for viral replication [79].

Considering the significant role of S protein in binding and entry to the host cells, we hypothesize that Omicron has AA variants located in S protein that can explain the high transmission rate and high immune evasion of Omicron. Using the DL query, we found 45 AA variants in Omicron (Fig. 7B), including 33 in S, 4 in pp1a, 3 in M, 2 in each of E and pp1b proteins, and 1 in N protein. Among these AA variants, many have been associated with changes in antibody recognition and consequently evasion. These include: S:E484K, S:N501Y, S:H69-, and S:144Y [76, 80–82] and are predominantly located on the N-Terminal Domain (NTD) of the S protein. The ribosomal binding domain of the S protein, however, has AA variants that affect binding to the S protein, and thus cell entry into SARS-CoV-2.

As further evidence of how inferencing with CIDO may be used to generate novel information, a Description Logic (DL)-query further found 18 AA variants in the Delta strain (Fig. 7C), including 10 in S protein, 3 in each of pp1b/nucleocapsid (N) proteins, and 1 in each of E/M/pp1a proteins. Compared to one AA variant (RG203KR) in the Omicron N protein, 3 AA variants (D377Y, D63G, and R203M) exist in the Delta N protein. The SARS-CoV-2 nucleocapsid (N) protein is an RNA-binding protein critical for viral genome packaging [83], and it is also involved in the coronavirus pathogenesis [84]. Delta was found to have reduced pathogenicity due to altered cell tropism but less transmissibility and immune evasion ability [74]. The fact of more variants in the N protein in the Delta variant likely contributes to the differences in transmission and virulence.

(3) CIDO-supported NLP for clinical and basic mechanism research

Given the large volumes of COVID-19 related text in the literature and in electronic health records (EHRs), it is impossible for humans to extract useful information from what is available in a short period of time. In such cases, Natural Language Processing (NLP) is required, and ontology can be used to significantly enhance the performance of NLP [85–87].

**Fig. 8:**
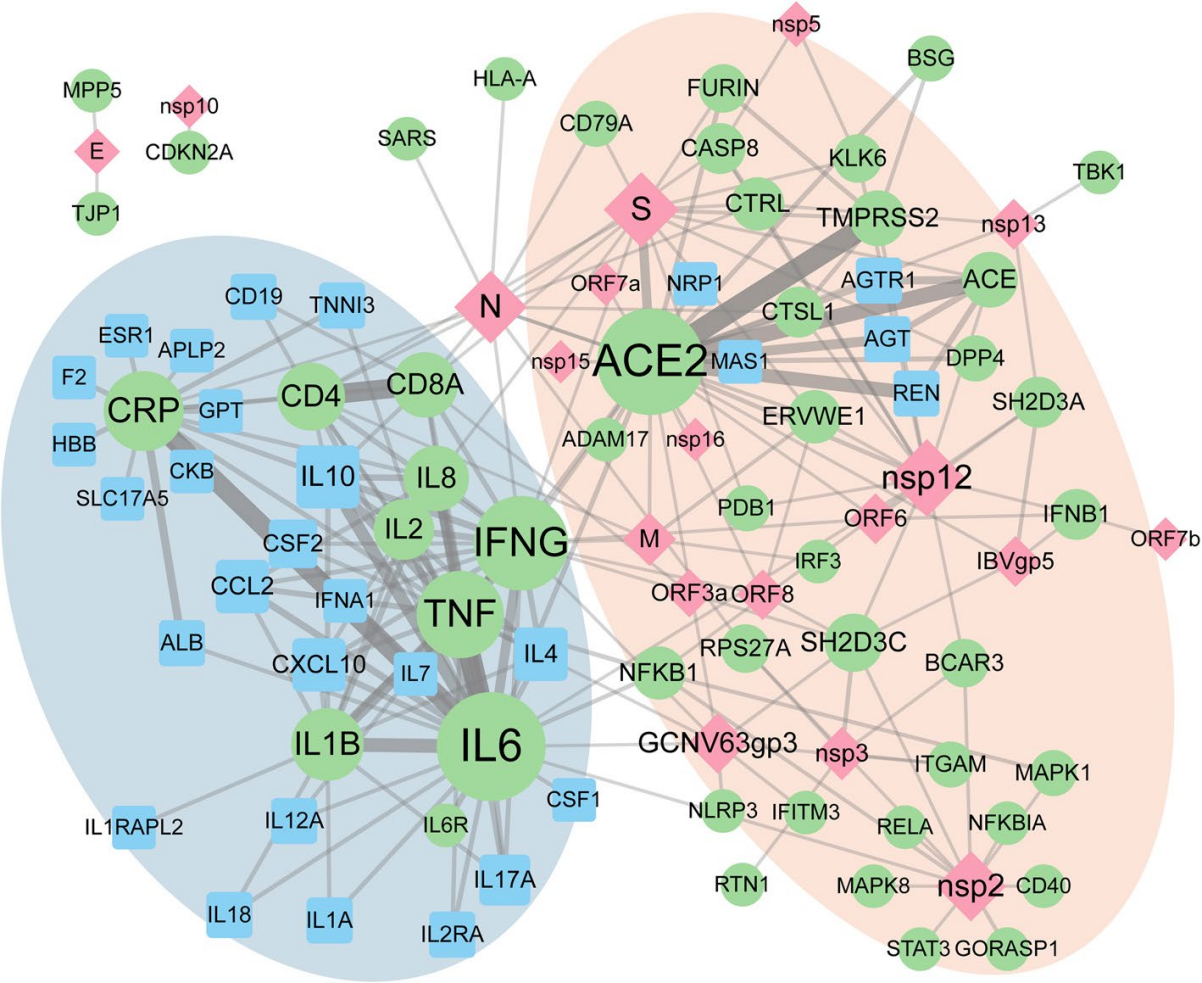
Host-SARS-CoV-2 gene-gene interaction network using SciMiner on the litCovid paper abstracts. Color represents the type of genes: pink (viral), green (host gene directly co-cited with pathogen genes at the sentence level), and cyan (host gene co-cited with the green host genes in at least 30 or more COVID-19 papers). Node size corresponds to the number of connections and edge thickness corresponds to the number of co-citing papers

Understanding how pathogen and host genes interact during infection can help to identify critical targets of intervention or prevention. In this connection CIDO has been used to support literature mining in relation to the molecular host-coronavirus interactions. SciMiner, our in-house tool for mining scientific literature using dictionary- and rule-based methods [88], has been integrated with biomedical ontologies and applied to the study of vaccine-associated gene interaction networks [89, 90]. Using coronavirus-specific genes and proteins covered in CIDO and in the Interaction Network Ontology (INO) [91], we have applied SciMiner to perform literature mining on host-coronavirus interactions. Figure 8 illustrates a gene-gene interaction network we constructed in February 2022 using a subset of SciMiner mining results from > 220 K COVID-19-related articles in LitCovid [92]. Two noticeable subclusters were identified, largely related to viral invasion (right), involving S protein and host genes such as ACE2 and TMPRSS2, and host immune response (left), including cytokines and proinflammatory responses. This network summarizes the major host-pathogen interactions of SARS-CoV-2 virus and host and can be further expanded with other vaccine components and serve as the foundation for mining analyses.

CIDO has also been used in EHR mining from clinical COVID-19 patient data in a recently proposed open NLP development framework that addresses the issues of NLP process heterogeneity and human factor variations [93]. A COVID-19 NLP algorithm was developed under the open NLP development framework. Specifically, the algorithm shared through the Open Health NLP (OHNLP) (https://github.com/OHNLP), was first used to identify COVID-19-associated terms including various signs and symptoms (e.g., cough and fever) from the EHR notes of COVID-19 patients from three N3C participant institutions, including Mayo Clinic, the University of Kentucky, and the University of Minnesota at Twin Cities. The identified terms were then mapped to the codes represented in CIDO. These codes are primarily imported from reference ontologies such as HPO and also cross-referenced to other ontologies or terminologies including UMLS [94], SNOMED-CT [95], MeSH [96], and MedDRA [97]. The usage of CIDO in the open NLP development framework supports the normalization of clinical NLP results from different N3C participant sites, leading to enhanced data integration and analysis in the future.

(4) CIDO-based machine learning and drug cocktail design for COVID-19 treatment

Anti-coronaviral drug design has been our first CIDO use case since the beginning of CIDO development [12] and we have systematically collected SARS/MERS/SARS-2 drug data for this purpose [52, 62], along with SARS-CoV-2 specific drug and host-coronavirus PPI data. These data have been used for machine learning and cocktail drug design as detailed below.

The drug-target linkage knowledge recorded in CIDO has been used to support candidate COVID-19 drug prediction (Smaili et al., WCO-2020: https://github.com/CIDO-ontology/WCO). Specifically, the OPA2Vec machine learning method [98] was used to transform the CIDO knowledge and other related information to vectors, which were further used as the input to predict the drugs targeted for COVID-19. Our preliminary study found that the drugs against SARS-CoV-2 exhibit patterns which overlap with but are yet different from experimentally identified drug candidates against SARS-CoV and MERS-CoV [99]. More detailed information is being produced and analyzed.

It is still a major challenge to develop a fully effective drug for COVID-19 treatment. Hundreds of chemicals and drugs have been experimentally verified to have anti-coronavirus function [52, 100]. Paxlovid from Pfizer, Molnupiravir from Merck, and Remdesivir [101] have been authorized for emergency usage; however, their effectivity remains low. In our previous paper, we proposed a host-coronavirus interaction (HCI) checkpoint cocktail that would interrupt the important checkpoints in the dynamic host-coronavirus interaction (HCI) network [62]. We hypothesized that such a cocktail of drugs would be more effective than the current COVID-19 vaccines. The question is then how to design this cocktail by identifying the HCI checkpoints and inferring how to interrupt them.

CIDO provides a solution to support rational HCI checkpoint classification and cocktail drug design as laid out in the above cocktail hypothesis. As earlier described and shown in Fig. 5, CIDO logically represents host-coronavirus protein-protein interactions (PPIs) and drugs targeting the viral or host proteins in the PPIs. Different proteins and PPIs have different roles in the HCI leading to disease outcomes. Major checkpoints such as the coronavirus entry (through S-ACE2 binding) and replication can then be defined. Interestingly, all the three drugs, Paxlovid (consisting of nirmatrelvir and ritonavir), Molnupiravir, and Remdesivir function by inhibiting enzymes responsible for coronavirus replication. Specifically, nirmatrelvir inhibits SARS-CoV-2 3C-like protease (i.e., nsp5) to stop the virus from replicating (Fig. 5), and ritonavir slows down nirmatrelvir’s breakdown to help keep it in the body for longer at higher concentrations. This 3C-like protease is responsible for cleaving polyproteins 1a and 1ab of SARS-CoV-2 into nonstructural proteins that are critical for viral replication. Molnupiravir and Remdesivir interfere with the action of RNA-directed RNA polymerase (RdRp), which is critical to viral replication as well. Based on our HCI checkpoint cocktail hypothesis, we would propose to include a drug targeting the viral entry, which can be used together with one of the existing drugs targeting the viral replication. A deeper CIDO-based study is ongoing to apply CIDO for the cocktail drug design.

**Fig. 9:**
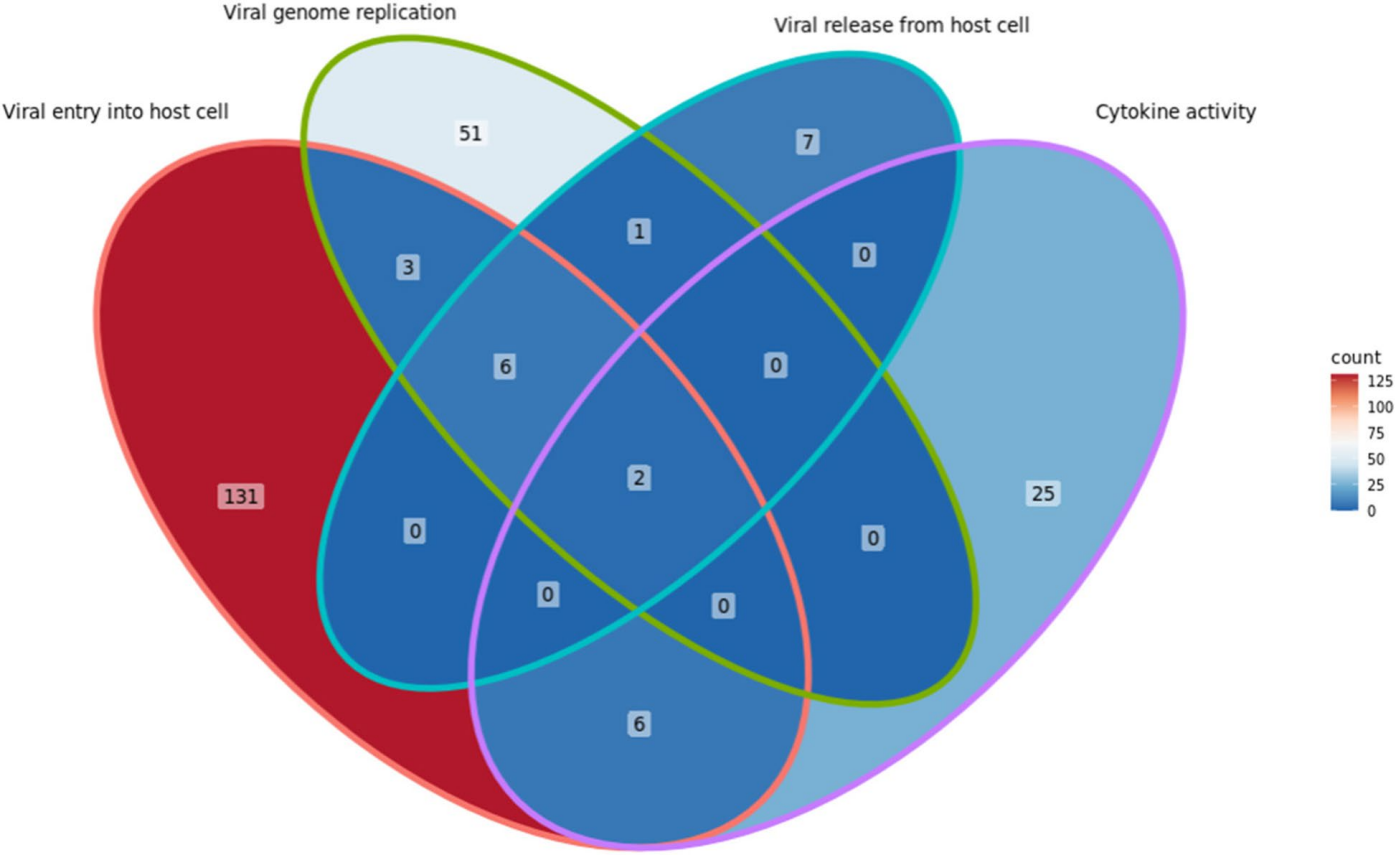
SARS-CoV-2 drug screening based on the drug cocktail strategy. A total of 232 drugs were identified to have their protein targets involving three coronavirus processes (i.e., viral entry, genome replication, and viral release) and/or host anti-coronaviral processes (i.e., cytokine activity). Two drugs (i.e., copper and artenimol) were shared to have protein targets involved in all four processes. The drug screening study was performed using the DrugXplore program (http://medcode.link/drugxplore/)

We (authors: ZW and YH) have implemented the cocktail strategy in our newly developed DrugXplore program (http://medcode.link/drugxplore/), which extends the OmicsViz program [8, 64]. Specifically, we used the host-coronavirus PPI and drug-target interaction data represented in CIDO and other resources such as BioGRID [102] to find drugs targeting different HCI processes. Figure 9 shows one result of our DrugXplore data analysis. A total of 232 drugs were identified to target three coronavirus processes (i.e., viral entry, genome replication, and viral release) and/or one host anti-coronaviral process (i.e., cytokine activity), and two drugs (i.e., copper and artenimol) were shared to target all four processes (Fig. 9). Many reports have found copper and artenimol and their derivative drugs are potent potential drugs for COVID-19 treatment [103–108].

## Discussion

This manuscript provides a comprehensive update on the development and applications of the community-based Coronavirus Infectious Disease Ontology (CIDO). Our study demonstrates that CIDO provides an ideal platform to integrate important data needed to research different coronavirus disease-related entities such as coronavirus and host taxonomy, coronavirus proteins and genes, protein variants, epidemiology, diagnostic medical devices, phenotypes, host-coronavirus interactions, drugs, and vaccines. The ontological representation of CIDO supports integrative representation and analysis of COVID-19 and other human coronavirus diseases. A visual evolution analysis of CIDO was performed. Five groups of CIDO applications are introduced, including COVID-19 data annotation and inferencing, Delta and Omicron comparisons, clinical data analysis, NLP, and COVID-19 drug repurposing.

Given intensive coronavirus research during the COVID-19 pandemic, we have conducted very active CIDO development and applications. Within a little more than 2 years, CIDO has grown to include over 10,000 terms, of which over 1500 terms are CIDO specific. Meanwhile, we acknowledge that CIDO has not yet covered all related areas and some areas of representation (e.g., host-coronavirus interactions, epidemiology, and public health) are still not fully covered. Many applications (e.g., machine learning, N3C data analysis, and drug repurposing design) have started but still need more time to achieve breakthrough outcomes. However, we have demonstrated many progresses and achievements in different applications in this manuscript.

An ongoing CIDO development effort is to actively model and represent various mechanisms of the molecular and cellular interaction between the hosts and coronaviruses. Such modeling will provide the foundation for our rational drug repurposing and vaccine development. For example, in our previous drug studies [52, 62], we extracted and analyzed the interactions between anti-coronavirus drugs and their target proteins. These anti-coronavirus drugs were identified to be effective against coronavirus infections in vitro or in vivo. It is likely that some of the drug targets participate in active host-SARS-CoV-2 interactions leading to severe COVID-19 disease outcomes. Deeper modeling and representation of the intricate host-virus-drug interactions would help us in better drug repurposing analysis.

We will continue our ontology harmonization effort to harmonize different COVID-19 related ontologies [14]. We will continue to update CIDO to handle the description of coronaviral variants. This is to account for immune escape and for previously designed treatments and vaccines losing efficacy. We will keep using CIDO as a platform to standardize different coronavirus-related metadata types and apply them for the standardization and enhanced analysis of specific conditions defined in different experimental and clinical studies, and how these conditions would affect the disease outcomes. We will also identify and develop more applications that implement CIDO for different purposes.

Being a community-based ontology, CIDO is committed to serving the community and to drawing on contributions from the community. CIDO is created to be open and freely available for use. It is an interoperable ontology that reuses and interlinks to existing ontologies and resources. We are always ready to accept new ideas and critiques. More researchers and developers are welcome to join our community-based effort to advance CIDO and its applications.

## Supplementary Information

**Additional file 1: Supplemental file 1.** Visualization of the Evolution of CIDO.

**Additional file 2: Supplemental Table 1.** Resources used for our coronavirus disease-related data collection.

**Additional file 3: Supplemental Table 2.** CIDO statistics including terms imported from major reference ontologies.

**Additional file 4: Supplemental Table 3.** Protein Ontology representation of SARS-CoV-2 proteins. Comparative information in RefSeq and UniProtKB is also provided.

## Data Availability

Related data, including the CIDO source code, is freely available on the GitHub website https://github.com/CIDO-ontology/cido.

## Abbreviations

AKI: Acute Kidney Injury
BFO: Basic Formal Ontology
ChEBI: Chemical Entities of Biological Interest
CIDO: Coronavirus Infectious Disease Ontology
DL query: Description Logics query
DO: Disease Ontology
DRON: Drug Ontology
GO: Gene Ontology
AO: Information Artifact Ontology
INO: Interaction Network Ontology
N3C: National COVID Cohort Collaborative
NCBITaxon: NCBI organismal classification
OBI: Ontology for Biomedical Investigations
OBO: The Open Biological and Biomedical Ontologies
OWL: Web Ontology Language
PR: Protein Ontology
RDF: Resource Description Framework
SPARQL: SPARQL Protocol and RDF Query Language
UBERON: Uberon multi-species anatomy ontology
