# Supplementary material for: A comprehensive update on CIDO: the community-based coronavirus infectious disease ontology
Source: J Biomed Semantics. 2022 Oct 21;13:25. doi: 10.1186/s13326-022-00279-z (PMC9585694; doi:10.1186/s13326-022-00279-z)
Supplement: Supplementary file 1 — Additional file 1: Supplemental file 1. Visualization of the Evolution of CIDO. [file 13326_2022_279_MOESM1_ESM.docx]

**Supplemental File 1. Visualization of the Evolution of CIDO**

**Method - Visualization of the Evolution of CIDO**

The paper presents a top-level hierarchical structure of CIDO, but this top-level view provides a very limited view of the full content of CIDO. To illustrate the challenge of capturing the whole content of CIDO, we present in Figure 1 in this file a layout of the full hierarchy of CIDO including all 10,853 concepts in the ontology. Due to the dynamic reality of COVID-19 research, CIDO is growing rapidly with new concepts frequently added to the ontology, generating new versions. Hence, an additional challenge is tracking the evolution of CIDO across its various versions over time. It is important for both curators and users of CIDO to be familiar with the types of concepts that were added between versions. For example, a user may wonder: have the types of concepts which were added during recent versions had an impact on my application or research?

In this appendix we will address those issues. In our previous work [1, 2], we introduced summarization networks of ontologies [3] called partial-area taxonomies. However, the partial-area taxonomy of the current version of CIDO contains as many as 2029 nodes and is too large for display. In [4] we introduced the Weighted Aggregate Partial Area Taxonomy (WAT) summarization networks, which enable a user to choose the number of nodes in the WAT by aggregating partial-areas with small number of concepts into an ancestor partial-area with large number of concepts (a user-defined parameter b distinguishes between small and large partial-areas).

In [5] we presented the WAT summarization network of an earlier version of CIDO with 5138 concepts (version 1.0.108). Since the diagram of this WAT was long and narrow, it was not fitting for display on a screen. Thus we introduced in [5] an alternative layout producing a diagram which is a better fit for the proportion of a screen(see [5] for details). In this paper we present the WAT for the current version of CIDO. We demonstrate how comparing the two WATs enables us to track the evolution of CIDO between the versions. In particular, one can easily observe which kinds of concepts were added to CIDO from an old version to the current version, and how many were added for each kind.

**Results - Visualization of the Evolution of CIDO**

To illustrate the challenge with the visual comprehension of CIDO, Figure 1 generated by the OAF tool [6], shows a color picture of the layout of its hierarchy. Small white boxes represent concepts, positioned in levels according to their longest hierarchical path to the root concept 'Thing' (calculated with Topological Sort). 'Thing' is located (as white dot) at the upper edge of the diagram. All the IS-A links represented by color lines emanating from a given level are colored with the same color. This way we see in the figure that some IS-A links from a concept in one level point to concepts in higher levels (closer to the root) rather than in the next level as most IS-A links do. This figure with its overwhelming appearance illustrates the challenges of visual comprehension of CIDO.

Comparing this figure to the similar figure for the older version (version 1.0.108) in [5], provides some global insight on the evolution between the two versions. The number of levels in Figure 1 is 40 while the number of levels in the corresponding Figure 2 in [5] was only 27. This shows that the additional concepts were not just added to existing levels, but few additional concepts are further away from the root 'Thing'. Such concepts are of high level of specification, since in an ontology each child along a hierarchical path is of higher specification than its parent. Indeed in Figure 1 we see at the bottom a long narrow  "tail" of 16 levels which did not exist in the corresponding figure of the earlier  version in [5]. According to our experience [7], the concepts in such a "tail" have a high likelihood of errors, due to their high level number. It is recommended to perform quality assurance [8] on those concepts, the number of which is relatively small.


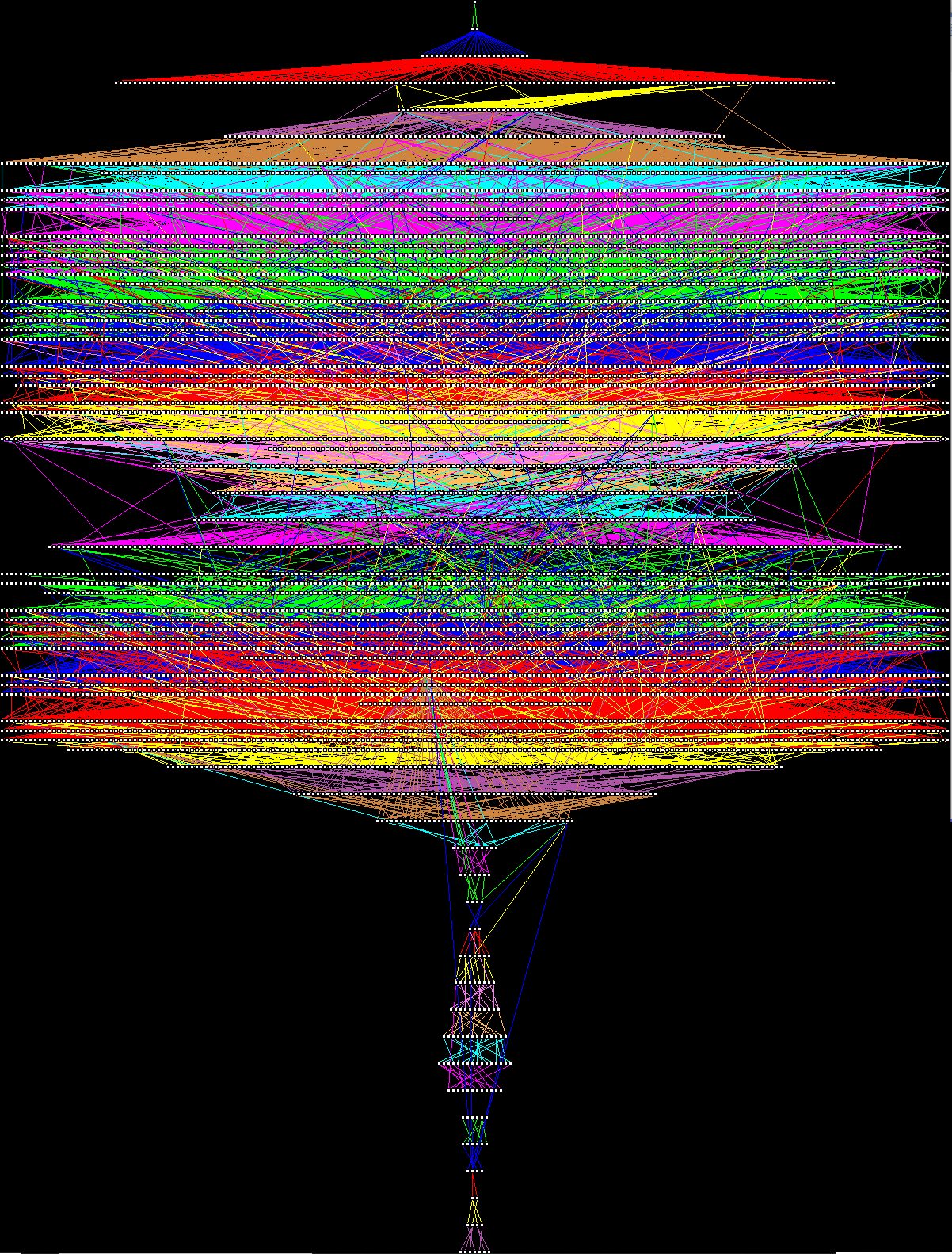


**Figure 1. Colorful layout of CIDO 10,853 concepts in layers according to their longest distance from the root.**

For a more detailed evolution analysis we will compare two weighted aggregate partial-area taxonomies (WATs) in Figures 2 and 3 designed with the OAF tool [6]. In Figure 2 we show the WAT for the older version which appeared in Figure 9 in [5]. In this figure there are 24 nodes each of which is an aggregate partial-area representing a major subject in CIDO. For example, the label for the node named 'process' is (301){11}[272] meaning that this aggregate partial-area consists of 301 process concepts, 272 of which are of the original partial-area rooted by the concept 'process' while the remaining 29 concepts were aggregated from 11 'small' descendant partial-areas. In this figure the bound is b=42 implying that all partial-areas with less than 42 descendant concepts are considered small and are aggregated into larger ancestor partial-areas. For more details on the design of WATs see [5]. The number of nodes (major subjects) of a WAT can be controlled by the choice of the bound b.

For assessing the evolution of CIDO between the two versions we designed the WAT in Figure 3 for the current version using the same bound b=42 and the same graphical notation. Figure 3 has 33 nodes. For example the label of the 'process' node is (1052){22}[995]. Comparing the two labels of 'process' in the two WATs enable us to track the evolution of the process concepts between these two versions. The partial-area 'process' itself grew from 272 to 995 concepts.

The number of small descendant partial-areas of 'process' which were aggregated into their ancestor 'process' partial-area grew from 11 to 22 and the number of their concepts grew from 29 to 57. To track the evolution of 'viral vaccine' we can compare similarly the labels of these nodes (at the bottom of the two WATs). The partial-area with this name grew by 6 new viral vaccines from 51 to 57, while the number of aggregated descendants partial areas grew from 7 to 9, each of which consists of a single concept. Similarly, one can track the evolution of concepts for each of the major subjects in CIDO which appear in both WATs. For example, the number of pharmaceutical preparations stayed the same at 88.

The more interesting phenomena in the evolution of CIDO is when new nodes appear or some old nodes disappear. For example the new WAT contains a node 'disease', which did not exist in the WAT of the older version, with a label (155){13}[130], which is a child of the node 'realizable entity.' Upon closer inspection we see that indeed the number in 'realizable entity' was reduced from 843 to 786. Apparently in the old version the number of disease concepts was lower than b=42 and when more disease concepts were added, 'disease' became a major subject by itself. Moreover, 'disease' has a child node 'viral infectious disease' since the partial-area with this name now has 42 concepts, just enough to make ‘viral infectious disease' a major subject of CIDO.

Similarly ‘material entity’ became smaller when its new child nodes 'organism', 'chemical entity' and 'gene' emerged from it. Furthermore 'gene' has a child node 'protein coding gene' with 504 concepts. This case is interesting because the 'gene' node has only 29 concepts, but due to its child node, its weight is more than 42 causing 'gene' to be shown in Figure 3 (For detailed explanation see [5]). A similar situation exists regarding the node 'vaccine' which has only 1 concept, but its weight is more than 42 due to its child node 'viral vaccine'. (The weight of a partial-area is defined as the number of descendant concepts of the root of this partial-area, including in descendant partial-areas.) Other new interesting nodes are 'COVID-19 vaccine', ‘carboxylic acid anion' and 'eukaryotic protein'. On the other hand, the node 'anatomical structure' which appeared in the WAT of the old version disappeared from the WAT of the new version, probably due to change in the modeling of some concepts.

As we see, the comparison of the nodes in the WATs of two versions of CIDO enables us to track what are the existing kinds of concepts and how many of them were added to CIDO, as well as what new kinds of concepts were added to CIDO during the evolution. Hence the WATs of two versions of CIDO enable curators and users to track the evolution of CIDO between these two versions. This technique is not unique for CIDO and enables tracking the evolution of other ontologies modeled with lateral relationships for which one can derive their weighted aggregate partial area taxonomies (WATs) by our OAF tool.


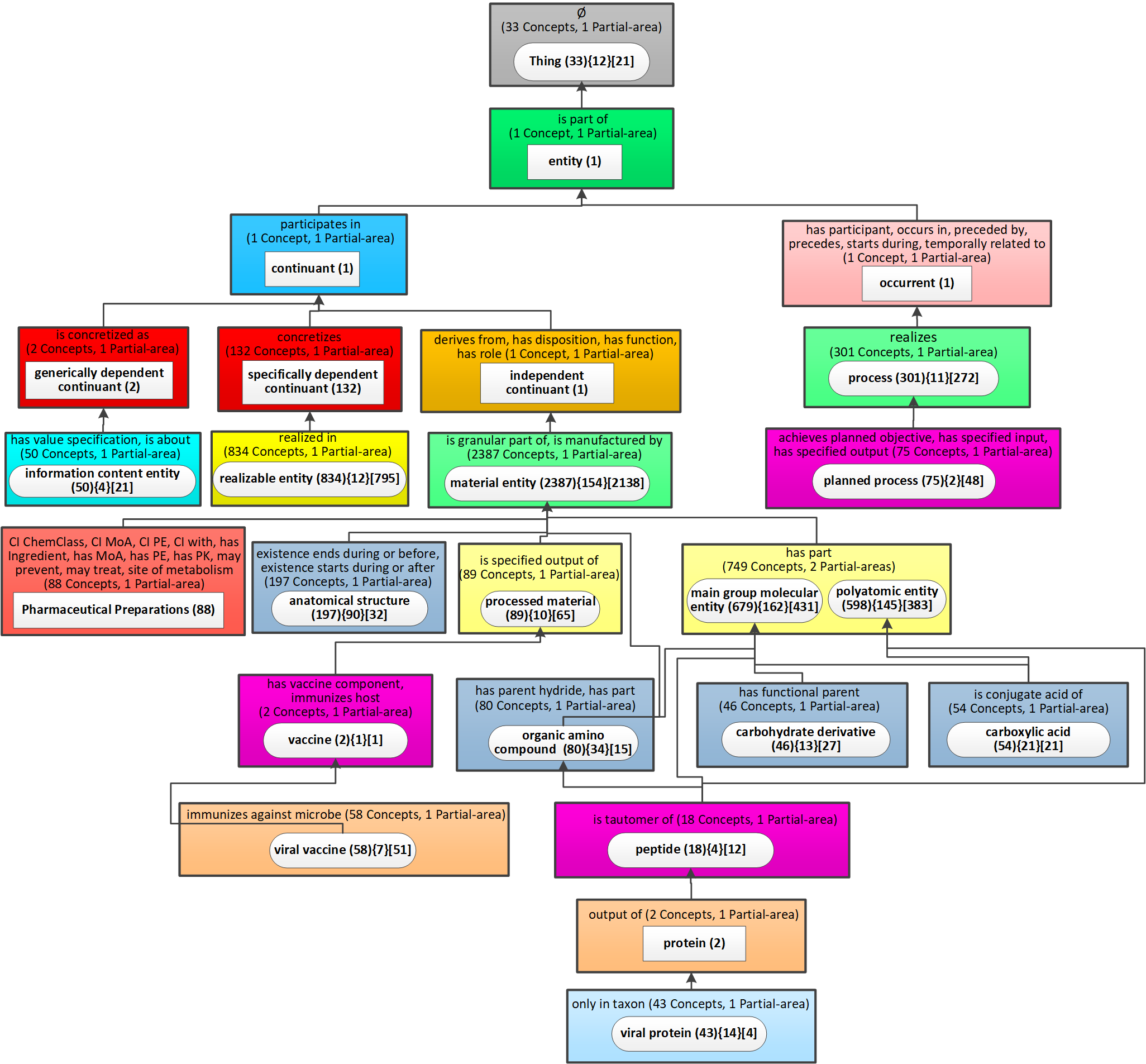


**Figure 2. The weighted aggregate taxonomy for CIDO (version 1.0.108) with 5,138 concepts (b = 42).**


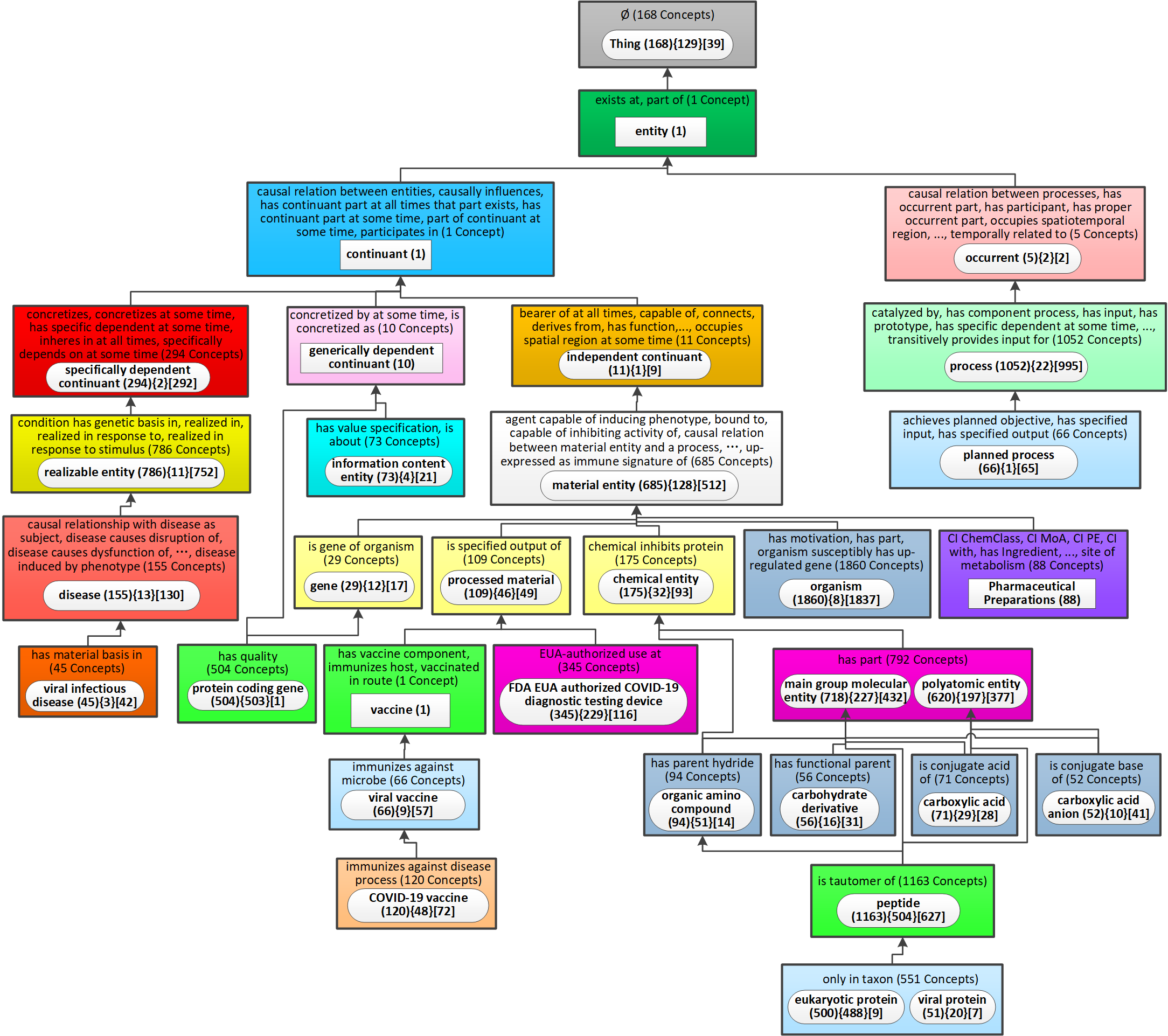


**Figure 3. The weighted aggregate taxonomy for CIDO (version 1.0.306) with 10,853 concepts (b = 42).**

**References**

1. Wang Y, Halper M, Min H, Perl Y, Chen Y, Spackman KA. Structural methodologies for auditing SNOMED. J Biomed Inform. 2007;40(5):561-81.

2. Min H, Perl Y, Chen Y, Halper M, Geller J, Wang Y. Auditing as part of the terminology design life cycle. J Am Med Inform Assoc. 2006;13(6):676-90.

3. Halper M, Gu H, Perl Y, Ochs C. Abstraction networks for terminologies: Supporting management of "big knowledge". Artif Intell Med. 2015;64(1):1-16.

4. Zheng L, Ochs C, Geller J, Liu H, Perl Y, De Coronado S, editors. Multi-layer Big Knowledge visualization scheme for comprehending neoplasm ontology content. 2017 IEEE International Conference on Big Knowledge (ICBK); 2017: IEEE.

5. Zheng L, Perl Y, He Y, Ochs C, Geller J, Liu H, et al. Visual Comprehension and Orientation into the COVID-19 CIDO Ontology. Journal of Biomedical Informatics. 2021.

6. Ochs C, Geller J, Perl Y, Musen MA. A unified software framework for deriving, visualizing, and exploring abstraction networks for ontologies. J Biomed Inform. 2016;62:90-105.

7. Min H, Zheng L, Perl Y, Halper M, De Coronado S, Ochs C. Relating Complexity and Error Rates of Ontology Concepts. More Complex NCIt Concepts Have More Errors. Methods Inf Med. 2017;56(3):200-8.

8. Halper M, Perl Y, Ochs C, Zheng L. Taxonomy-Based Approaches to Quality Assurance of Ontologies. J Healthc Eng. 2017;2017:3495723.
