# Supplementary material for: A comprehensive update on CIDO: the community-based coronavirus infectious disease ontology
Source: J Biomed Semantics. 2022 Oct 21;13:25. doi: 10.1186/s13326-022-00279-z (PMC9585694; doi:10.1186/s13326-022-00279-z)
Supplement: Supplementary file 2 — Additional file 2: Supplemental Table 1. Resources used for our coronavirus disease-related data collection. [file 13326_2022_279_MOESM2_ESM.docx]

**Supplemental Table 1. Resources used for our coronavirus disease-related data collection.**

| **Topic** | **Resource** | **URL** |
| --- | --- | --- |
| Literature | PubMed | <https://pubmed.ncbi.nlm.nih.gov/> |
|  | PubMed Central | <https://www.ncbi.nlm.nih.gov/pmc/> |
| Virus variations | GISAID | <https://www.gisaid.org/> |
|  | NextStrain | <https://nextstrain.org/> |
|  | PANGO | <https://outbreak.info/> |
|  | WHO | <https://www.who.int/en/activities/tracking-SARS-CoV-2-variants/> |
| Drugs and drug targets | DrugBank | <https://www.drugbank.com/> |
| Chemicals | ChEBI | <https://www.ebi.ac.uk/chebi/> |
| Diagnostic testing | FDA EUA diagnostic testing | <https://www.fda.gov/medical-devices/coronavirus-disease-2019-covid-19-emergency-use-authorizations-medical-devices/in-vitro-diagnostics-euas> |
|  | AdveritasDx | <http://adveritasdx.com/> |
|  | LOINC In Vitro Diagnostic (LIVD) Test Code Mapping for SARS-CoV-2 Tests | <https://www.cdc.gov/csels/dls/sars-cov-2-livd-codes.html> |
