# Supplementary material for: A comprehensive update on CIDO: the community-based coronavirus infectious disease ontology
Source: J Biomed Semantics. 2022 Oct 21;13:25. doi: 10.1186/s13326-022-00279-z (PMC9585694; doi:10.1186/s13326-022-00279-z)
Supplement: Supplementary file 3 — Additional file 3: Supplemental Table 2. CIDO statistics including terms imported from major reference ontologies. [file 13326_2022_279_MOESM3_ESM.docx]

**Supplemental Table 2. CIDO statistics including terms imported from reference ontologies.**

| **Domain** | **Ontology** | **# of terms** |
| --- | --- | --- |
| Taxonomy of viruses | NCBI Taxonomy Ontology (NCBITaxon) | 1810 |
| Phenotypes | Human Phenotype Ontology (HPO) | 246 |
| Diseases | Human Disease Ontology (DOID) | 102 |
| Infectious Diseases | Infectious Disease Ontology (IDO) | 23 |
| Anatomy | Uber-anatomy ontology (UBERON) | 122 |
| Proteins | Protein Ontology (PR) | 1855 |
| Genes and genomes | Ontology of Genes and Genomes (OGG) | 51 |
| Biological processes, Molecular functions, and cellular components | Gene Ontology (GO) | 564 |
| Chemical compounds | Chemical Entities of Biological Interest (ChEBI) | 2009 |
| Vaccines | Vaccine Ontology (VO) | 302 |
| Drugs | Drug Ontology (DrON) | 123 |
|  | National Drug File - Reference Terminology  (NDF-RT) | 1,473 |
| Adverse Events | Ontology of Adverse Events (OAE) | 88 |
| Lab measurements | Ontology for Biomedical Investigations (OBI) | 267 |
| Biological and Clinical Statistics | Ontology of Biological and Clinical Statistics (OBCS) | 11 |
| Relations | Relation Ontology (RO) | 210 |
| Precision medicine | Ontology of Precision Medicine Investigation (OPMI) | 133 |
| **CIDO-specific** | **Coronavirus Infectious Disease Ontology (CIDO)** | **1,514** |
| Miscellaneous |  | 1,166 |
| Total |  | **12,069** |

***Note:*** The terms include classes, object properties, datatype properties, annotation properties, and instances. The counts were reported from Ontobee Statistics (<https://www.ontobee.org/ontostat/CIDO>) as of May 16, 2022.
